# Stability of Diazoxide in Extemporaneously Compounded Oral Suspensions

**DOI:** 10.1371/journal.pone.0164577

**Published:** 2016-10-11

**Authors:** Mihaela Friciu, Sarra Zaraa, V. Gaëlle Roullin, Grégoire Leclair

**Affiliations:** Faculté de pharmacie, Université de Montréal, Montréal, QC, Canada; Institute of medical research and medicinal plant studies, CAMEROON

## Abstract

The objective of this study was to evaluate the stability of diazoxide in extemporaneously compounded oral suspensions. Oral suspensions of diazoxide 10 mg/mL were prepared from either bulk drug or capsules dispersed in either Oral Mix or Oral Mix Sugar Free. These suspensions were stored at 5°C and 25°C/60%RH in bottles and oral syringes for a total of 90 days. At predetermined time intervals, suspensions were inspected for homogeneity, color or odor change; the pH was measured and the concentration of diazoxide was evaluated by ultraviolet detection using a stability-indicating high pressure liquid chromatography method. All preparations were demonstrated to be chemically stable for at least 90 days.

## Introduction

Diazoxide is a potassium channel activator that increases the diffusion of potassium ions through plasma membranes and induces a local relaxation in smooth muscles. This vasodilatory effect is useful for the treatment of acute and malignant hypertension [1]. Diazoxide is also used to treat hypoglycemia [2]. Indeed, it is also a highly potent beta cell K_ATP_ channel opener. It causes an hyperpolarization of pancreatic beta cells and inhibits the secretion of insulin [3].

According to the Canadian product monograph of Proglycem (diazoxide 100 mg capsules) [4], this product is indicated in adults for the management of hypoglycemia due to hyperinsulinism associated with inoperable islet cell adenoma or carcinoma as well as extrapancreatic malignancy. In infant and children, Proglycem is indicated for leucine sensitivity, islet cell hyperplasia, nesidioblastosis, extrapancreatic malignancy, islet cell adenoma, or adenomatosis [4]. Recommended doses in adult and children are between 3 and 8 mg/kg [4]. In infant and newborn the recommended doses are between 8 and 15 mg/kg [4]. In all cases, the daily dose should be divided in two or three equal doses every 8 to 12 h [4]. Diazoxide is a known teratogen [5–6] and should not be administered to women of child-bearing age except in life-threatening situations [4].

Diazoxide is used in the pediatric population since the 1960s to manage hypoglycemia [7–8], and von Gierke’s disease [9]. It is still considered the first line of treatment for hyperinsulinaemic hypoglycemia of infancy [10–11].

In the USA, diazoxide is available as a 50 mg/mL oral suspension (Proglycem, NDA 016453, Teva Pharmaceuticals USA) [12]. This chocolate-mint flavored suspension is intended to be stored at controlled room temperature. In Canada, diazoxide is only available as 100 mg capsules (Proglycem, DIN 00503347, Merck Canada). As no stability data has been published on extemporaneously compounded diazoxide oral liquid formulations, few options remain for using this compound in the pediatric population in this country. It is indeed very difficult to achieve precise dosage (8 to 15 mg/kg/day, divided in three doses) in infant and newborn using 100 mg capsules.

According to the United State Pharmacopeia (USP), it is required to determine a date after which a compounded preparation shall not be used [13]. In the absence of stability study, the USP recommends a beyond use date of 14 days for water-containing oral preparations stored at controlled cold temperature [13]. However, this default date is questionable when the stability of a given preparation is unknown.

Eight recent reports on the stability of compounded oral liquid preparations listed in PubMed and coming from different scientific journals were reviewed to determine the current standard for such studies [14–21]. For all these studies, the concentration of the drug at different time points was reported as a percentage of the initial concentration. This concentration should be not less than 90% of the initial concentration to be considered acceptable. All these studies were performed by HPLC-UV, but one that was performed by HPLC-MS/MS. Six of these eight studies included the evaluation of pH; two informally evaluated the taste, one evaluated the viscosity and one performed microbiological testing. Studies are usually performed over a period of three months (five studies), two studies lasted one month and one study lasted four months. The total number of time points including initial varied between four and twelve. Studies were all conducted under refrigerated and controlled room temperature conditions with the exception of one study that was conducted only at controlled room temperature. Typically, the design was such that the degradation was replicated two or three times for each tested conditions (five studies). That could be achieved by preparing multiple batches (two studies) or by dividing a single batch into multiple containers (three studies); three studies did not replicate the degradation conditions. The number of replicated analyses or sample preparation was not reported except for two studies. In one case, injections were duplicated. In another case, sample preparation was repeated six times from the same sample as degradation conditions were not replicated. Six studies evaluated the linearity of the HPLC method, while five studies evaluated intraday and interday variability. Specificity of the HPLC method was evaluated with regards to the other excipients for at least five of the eight studies. Forced degradation of the preparations was also performed to evaluate the specificity of the HPLC method with regard to potential degradation products for five of the eight studies.

The objective of this study was first to develop and validate a stability-indicating HPLC-UV method for diazoxide and then to evaluate the stability of simple preparations of this drug compounded in Oral Mix and Oral Mix Sugar Free (SF). The suspensions were prepared from the bulk diazoxide powder as well as from diazoxide capsules. Oral Mix and Oral Mix SF are frequently used, dye-free, cherry flavoured oral suspending vehicles from Medisca Pharmaceutique Inc.

## Materials and Methods

### Reagents and chemicals

Diazoxide, glycerin, 50 mL amber PET bottles with black phenolic caps, 1 mL amber plastic oral syringes with tip caps, Oral Mix and Oral Mix SF vehicles used in this study were provided by Medisca Pharmaceutique Inc. (QC, Canada). All chemicals used are at United States Pharmacopeia standards when applicable. Diazoxide 100 mg capsules (Proglycem, Merck, Canada) were also used. Acetonitrile and dimethylsulfoxide were HPLC grade and were obtained from Fisher Scientific (QC, Canada). Potassium phosphate monobasic was purchased from JT Baker (NJ, USA). Purified water was deionised using a MilliQ system to 18 MΩ resistivity.

### Preparations compounded from bulk diazoxide powder

Bulk diazoxide powder (2 g) was accurately weighed and mixed in a mortar with glycerin (3 mL) until forming a smooth paste. Oral Mix or Oral Mix SF were then added incrementally to form a uniform suspension having a target concentration of 10 mg/mL (*q*. *s*. *ad* 200 mL).

### Preparations compounded from diazoxide capsules

Similarly, diazoxide suspensions (10 mg/mL) were compounded from capsules with both Oral Mix and Oral Mix SF. The content of diazoxide capsules (25 × 100 mg capsules) were pulverized using a pestle in a mortar. The powder was mixed with a small amount of vehicle (10 mL) to form a homogeneous paste. Additional vehicle was added in increments, up to the required final volume (*q*. *s*. *ad* 250 mL) and thoroughly mixed to form a uniform suspension.

### Design of the stability study

The four preparations were packaged in 50-mL amber PET bottles with black phenolic caps (filling volume of 25 mL) and 1-mL amber plastic oral syringes with tip caps (filling volume of 1 mL). A total of six bottles and 48 syringes were conditioned for each preparation; three bottles and 24 syringes were stored under refrigeration (5 ± 2°C), while the same number of containers were stored at controlled room temperature (25 ± 2°C / 60 ± 5% RH–Thermo Scientific, Forma Environmental Chamber, OH, USA).

At predetermined time points (0, 7, 14, 30, 45, 60, 75 and 90 days), an aliquot was sampled from each bottle (1 mL) and three syringes were retrieved for each preparation at each temperature condition. Prior to sampling, bottles and syringes were vigorously shaken (10 s) and the suspensions inspected for homogeneity, color and odor changes. In the case of syringes, the organoleptic properties were verified after the transfer of suspensions into 1.5-mL centrifuge tubes. Diazoxide concentration was evaluated using a validated stability-indicating HPLC-UV method, while pH was measured using a pH meter (Hanna Instruments pH 211, QC, Canada). Stability was defined as a recovery of not less than 90.0% of the initial concentration and a variation of less than 0.5 unit of pH.

### Sample preparation for HPLC injection

Preparation (50 μL, 10 mg/mL nominal concentration) was first transferred into a 1.5-mL centrifuge tube. A dimethylsulfoxide:acetonitrile mixture (1:3 v/v, 450 μL) was then added to dissolve diazoxide and precipitate other excipients. The tube was vigorously mixed using a vortex (20 s) and then centrifuged (12,000 g, 15 min). Supernatant (20 μL) was transferred into another 1.5-mL centrifuge tube and an acetonitrile:water solution (1:4 v/v, 400 μL) was added. This tube was vigorously mixed using a vortex (10 s) and transferred to a 96-well plate for HPLC analysis.

### Forced degradation experiments

A stock diazoxide suspension (10 mg/mL) was prepared in Oral Mix as described above. Aliquots of this suspension (0.5 mL) were subjected to forced degradation in presence of water (0.5 mL), aqueous NaOH (0.5 mL, 1 M), aqueous HCl (0.5 mL, 1 M) and aqueous H_2_O_2_ (0.5 mL, 3% v/v). These mixtures were then heated to 60°C for 65 h. A mixture with H_2_O_2_ (0.5 mL, 30% v/v) was also heated at 60°C for 20 h. Aliquots of acidic and alkaline suspensions (100 μL) were first neutralized with aqueous sodium hydroxide 1 M or aqueous hydrochloric acid 1 M (50 μL) respectively, and then diluted using a dimethylsulfoxide:acetonitrile mixture (1:3 v/v, 350 μL). Water and peroxide suspensions were directly diluted using the dimethylsulfoxide:acetonitrile mixture (1:3 v/v, 400 μL). All these solutions were vortexed (20 s) and centrifuged (9400 g, 10 min). Supernatants (20 μL) were recovered and diluted in an acetonitrile:water solution (1:4 v/v; 380 μL) to achieve a nominal concentration of 50 μg/mL (prior degradation) and assayed using HPLC-UV method.

### HPLC-UV method

The quantification of the drug was performed using a reversed-phase HPLC system (Shimadzu Prominence UFLC) comprising an LC-20AD binary pump, a DGU-20A5 solvent degasser, a SPD-M20A multiple wavelength photodiode array detector (PDA), a SIL-20AC HT refrigerated autosampler and a CTO-20AC column oven.

The flow rate was 0.4 mL/min and the injection volume was 10 μL. The UV detector wavelength was set at 265 nm. The separation was performed at 40°C on a Phenomenex Luna C18 (3.0 x 100 mm, 3 μm) column. An isocratic elution of 7 min was used where the composition of the mobile phase was a volumetric mixture of phosphate aqueous solution (potassium phosphate, monobasic, 10 mM, 25%) and acetonitrile (75%).

### HPLC-UV method validation

Linearity of the method was verified by constructing a calibration curve in both tested vehicles (Oral Mix and Oral Mix SF). Stock diazoxide suspensions were freshly prepared as described above at 10 mg/mL using both vehicles. The sample preparation for HPLC injection procedure was then adapted to obtain the following standard solutions: 25, 50, 75 and 100 μg/mL. These standard solutions were injected in triplicate in the HPLC system to calibrate the method and verify its linearity.

Intraday variability was calculated for each concentration using the results of the first calibration. Similarly, interday variability was calculated from the results of first calibration as well as the calibration performed at the following two stability time points.

Specificity of the method was evaluated by injecting the stress degradation samples. As a photodiode array detector was used, it was also possible to evaluate the peak purity index of the diazoxide peak of each chromatogram during the stability study. Peak purity index is an indication of the similarity of the chromatograms obtained between 235 and 295 nm. An acceptance specification of not less than 0.9999 was used to confirm the absence of coelution.

### Statistical analyses

Results are reported as means ± standard deviations unless otherwise noted. Diazoxide concentrations during the stability study are reported as percentages of the initial concentration.

## Results

### Assay validation

The linearity of the analytical method was confirmed from the calibration curves (r^2^ > 0.9999). Highest intraday coefficients of variation were 0.14% for standards prepared using Oral Mix and 0.08% for standards prepared using Oral Mix SF. Highest interday coefficients of variation were 0.38% for standards prepared using Oral Mix and 0.96% for standards prepared using Oral Mix SF. No matrix effect was observed in the calibration of this method, as the difference between the calibration factor obtained for standards prepared using Oral Mix (373,935 area unit/mg/mL) and the calibration factor obtained for standards prepared using Oral Mix SF (373,261 area unit/mg/mL) was comparable to the intraday variability of the method.

**Fig 1:**
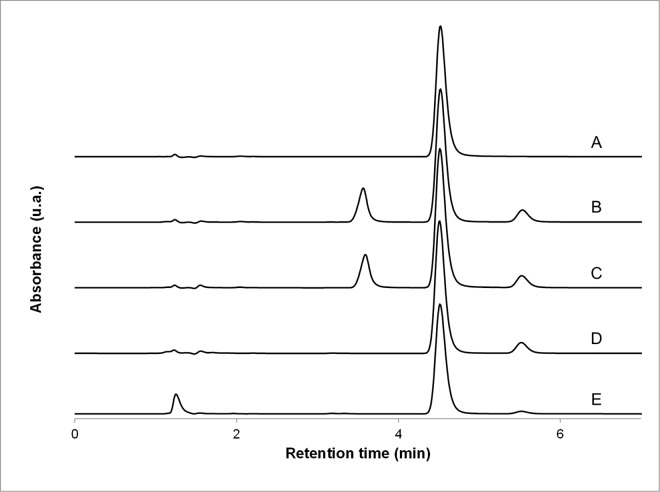
Representative chromatograms. (A) Diazoxide standard solution in acetonitrile:water (1:4 v/v); (B) diazoxide suspension prepared form bulk drug using Oral Mix vehicle; (C) diazoxide suspension prepared form bulk drug using Oral Mix SF vehicle; diazoxide suspension in Oral Mix after mild peroxide degradation conditions (D, 3.0%, 60°C, 65 h) and harsh peroxide degradation conditions (E, 30.0%, 60°C, 20 h). All sample preparations for injection resulted in nominal diazoxide concentrations of 50 μg/mL.

As no significant degradation was observed using the neutral, acidic, alkaline and 3.0% peroxide force degradation conditions (60°C, 65 h), harsher peroxide conditions were evaluated (30.0%, 60°C, 20 h). Under these harsher conditions, diazoxide recovery was 83% of the initial concentration. Fig 1 shows representative chromatograms of diazoxide in the absence of vehicle (A), in the presence of Oral Mix (B), in the presence of Oral Mix SF (C), submitted to 3.0% oxidative forced degradation (D) and submitted to 30.0% oxidative forced degradation (E). No interference was observed between the main diazoxide peak and other peaks produced by excipients, impurities or degradation products. The diazoxide peaks of all chromatograms obtained from the forced degradation studies were submitted to peak purity analysis. In all cases, the purity index was not smaller than 0.9999. These results confirmed that this HPLC-UV method was stability-indicating.

### Stability study

**Fig 2:**
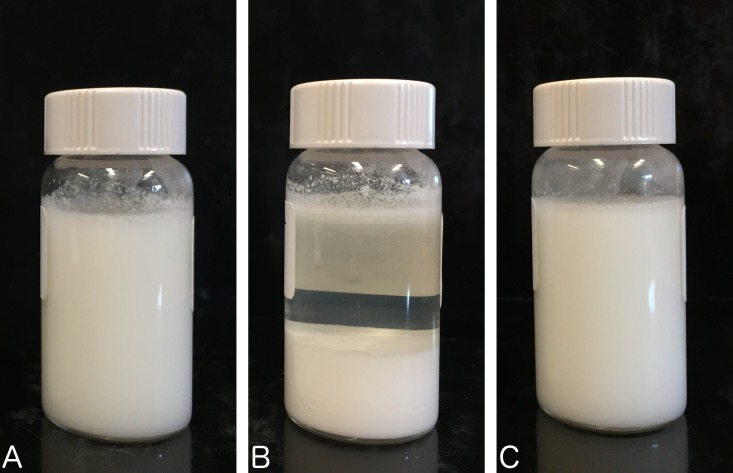
Representative example of the sedimentation and redispersion of the diazoxide suspensions. (A) The diazoxide suspension in Oral Mix was placed in a 20-mL clear vial; (B) this vial was stored at 5°C for 6 days (B); and then (C) gently shaken for 10 s.

At each pre-determined time point, preparations were shaken by hand prior to sampling in order to obtain homogeneous suspensions. A redispersible sedimentation, as exemplified in Fig 2, was observed after 7 days in plastic oral syringes. All suspensions maintained their initial opaque white color and no organoleptic changes were observed during the whole study. Also, the pH of all sample remained between 4.4 and 4.5 at all studied conditions during the whole study.

**Fig 3:**
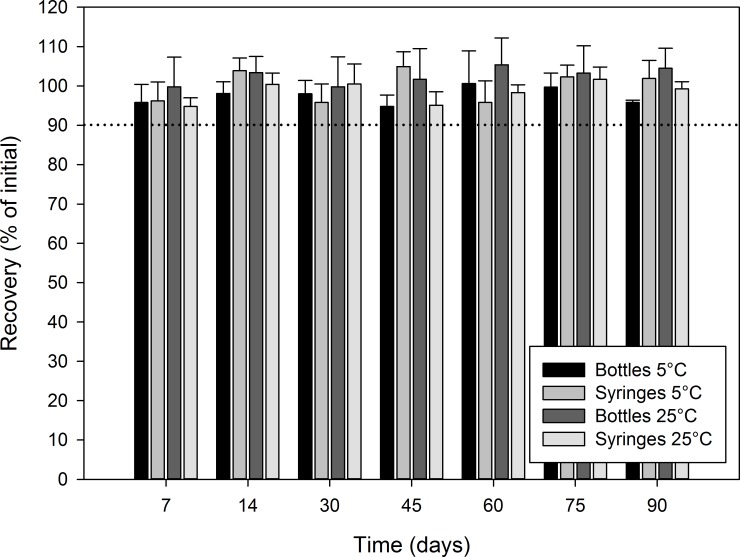
Chemical stability of diazoxide 10 mg/mL suspensions prepared from bulk drug powder in Oral Mix. The recovery as percentage of the initial concentration is reported as a function of time under different conditions: (black) bottles at 5°C, (light grey) syringes at 5°C, (dark grey) bottles at 25°C, and (white) syringes at 25°C. The initial concentration was 10.76 ± 0.05 and 9.25 ± 0.25 mg/mL for the suspensions stored in bottles and syringes, respectively.

**Fig 4:**
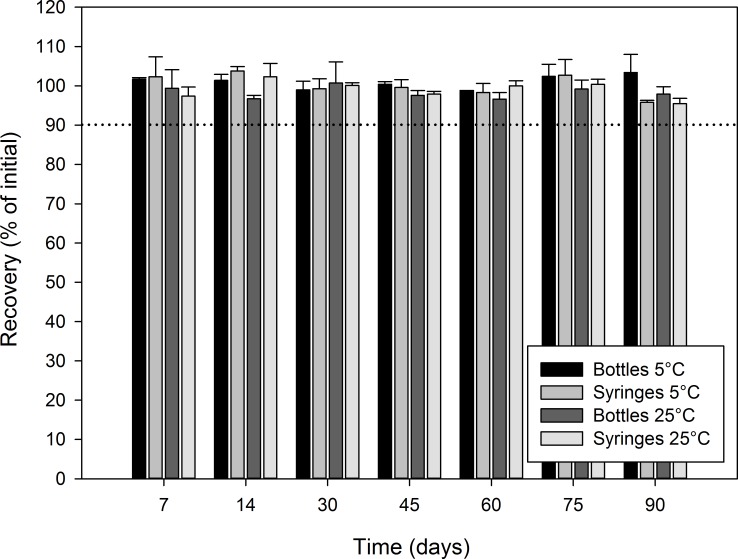
Chemical stability of diazoxide 10 mg/mL suspensions prepared from capsules in Oral Mix. The recovery as percentage of the initial concentration is reported as a function of time under different conditions: (black) bottles at 5°C, (light grey) syringes at 5°C, (dark grey) bottles at 25°C, and (white) syringes at 25°C. The initial concentration was 10.19 ± 0.06 and 10.01 ± 0.14 mg/mL for the suspensions stored in bottles and syringes, respectively.

**Fig 5:**
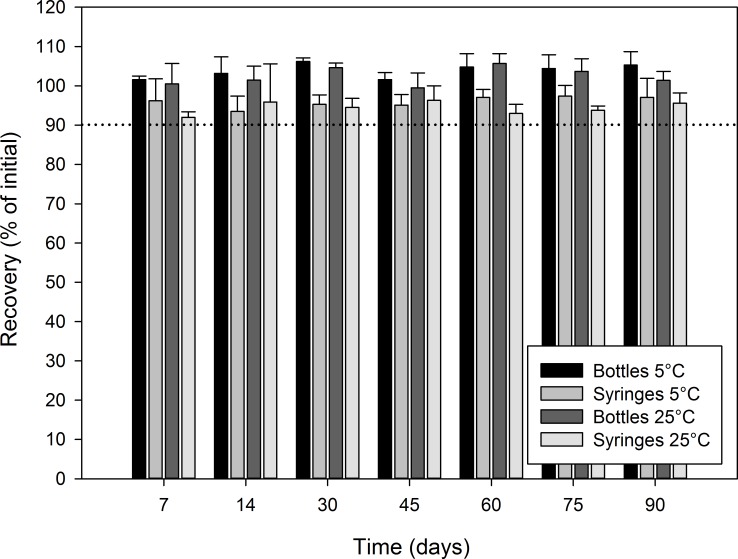
Chemical stability of diazoxide 10 mg/mL suspensions prepared from bulk drug powder in Oral Mix SF. The recovery as percentage of the initial concentration is reported as a function of time under different conditions: (black) bottles at 5°C, (light grey) syringes at 5°C, (dark grey) bottles at 25°C, and (white) syringes at 25°C. The initial concentration was 10.04 ± 0.18 and 9.98 ± 0.31 mg/mL for the suspensions stored in bottles and syringes, respectively.

**Fig 6:**
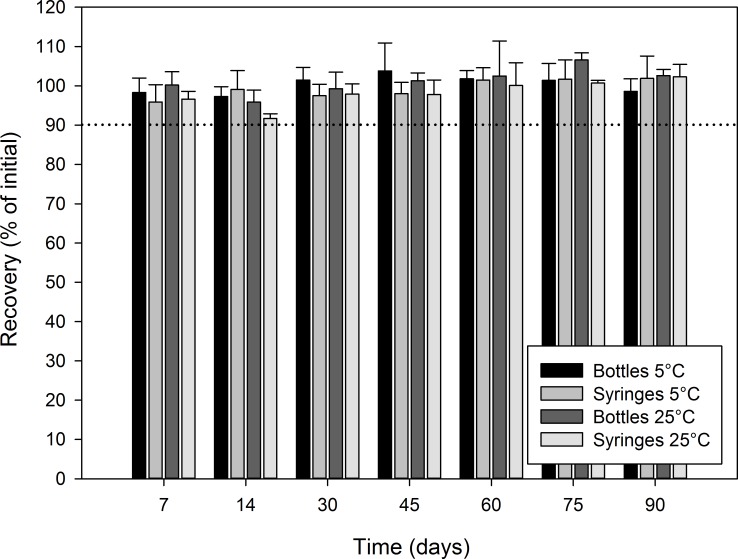
Chemical stability of diazoxide 10 mg/mL suspensions prepared from capsules in Oral Mix SF. The recovery as percentage of the initial concentration is reported as a function of time under different conditions: (black) bottles at 5°C, (light grey) syringes at 5°C, (dark grey) bottles at 25°C, and (white) syringes at 25°C. The initial concentration was 10.22 ± 0.17 and 10.05 ± 0.08 mg/mL for the suspensions stored in bottles and syringes, respectively.

Figs 3–6 report the recovery of diazoxide relative to initial for each stability time point. In all cases, the recovery relative to the initial concentration was not less than 90.0%, indicating that diazoxide preparations were stable at all studied conditions for at least 90 days: oral syringe vs. bottle; 5°C vs. 25°C; Oral Mix vs. Oral Mix SF; and, bulk drug vs. from capsules.

## Discussion

During the development of the diazoxide preparations, it was noted that uniform suspensions were more difficult to prepare from bulk powder than from capsules. On the one hand, the bulk powder was highly crystalline and could not be properly wetted using the oral vehicles. It required the use of a small amount of glycerine as a wetting agent. On the other hand, the capsule content was a fine pulverulent powder comprising hydrophilic lactose monohydrate in addition to diazoxide [4]. The use of glycerin was not required when the suspensions were prepared from capsules as it was much easier to disperse.

In this stability study, each preparation was divided between multiple containers to ensure replication of degradation conditions (three bottles and three oral syringes per time point for each temperature condition). The study was conducted over a total period of 90 days at 5°C and 25°C with a total number of eight time points. The pH as well as drug concentrations were determined by HPLC-UV using duplicated injections at each time point. The HPLC-UV method was validated by the evaluation of its linearity, intraday and interday variability as well as specificity with regards to excipients and potential degradation products. This was in line with current reported practice for such studies [14–21].

During the stability study, the recovery of all the samples remained within specifications (not less than 90.0% of initial concentration). Nonetheless, some variability was observed in the stability study as calculated recoveries fluctuated above and below 100%. At least three factors can contribute to a recovery different than 100% of initial during the stability studies: (1) chemical degradation of diazoxide during the study; (2) analytical error of the initial HPLC assay; and (3) analytical error of the HPLC assay at a given time point. Factor 1 is not random and should result in a negative correlation between the recoveries and time points. Factors 2 and 3 are random. However, the analytical error on the initial concentration will cause a systematic error in the calculated recovery for all further time points for a given preparation. Factor 3 should be random between time points. Linear regression of the recovery as a function of time was calculated for each of the 16 studied conditions. No correlation of recovery with time could be statistically demonstrated as the regression coefficients (r^2^) were comprised between 0.002 and 0.5. Assuming that recovery was not affected by time, then factors 2 and 3 become the major component of the observed variability during the stability study. The significance of factors 2 and 3 can then be evaluated by calculating the average of the recoveries at all time points for each of the 16 studied conditions. In the absence of degradation, the average recovery should be an indication of factor 2 (the accuracy of the initial HPLC assay) and its standard deviation should be an indication of factor 3 (the random analytical error). Average recoveries were comprised between 97.5 ± 2.2% and 102.6 ± 2.2% for each studied condition, with three notable exceptions: suspension prepared from bulk drug and Oral Mix SF and stored in bottles at 5°C (103.9 ± 1.8%), suspension prepared from bulk drug in Oral Mix SF and stored in oral syringes at 5°C (96.0 ± 1.4%) and suspension prepared from bulk drug in Oral Mix SF and stored in oral syringes at 25°C (94.4 ± 1.6%). An average recovery above 100% suggests that the initial assay was inferior to the real value, while an average recovery below 100% suggests that the initial assay was superior to the real value. When comparing stability results obtained for suspensions prepared from bulk powder in Oral Mix SF and stored in bottle at 5°C (average of 103.9%) with the same preparation stored in oral syringes at 5°C (average of 94.4%), the apparent difference in the recoveries is mostly caused by the analytical error of the HPLC analyses at time zero (underestimated for the suspension stored in bottle and overestimated for the suspension stored in syringes).

Approved diazoxide suspensions and capsules are required to contain not less than 90.0% and not more than 110.0% of the labeled amount of diazoxide [22–23]. During this study, the absolute concentration of diazoxide was always within these limits except for the suspension prepared from bulk drug and stored in oral syringes at 5°C and 25°C where five out of 16 evaluations were below 90.0% of label claim, with a lowest value of 87.7% of label claim. Nonetheless, the absolute concentration was within USP specification at time zero and at the end of the study, suggesting a real initial concentration slightly above 90.0% of label claim. Intermediate time points below 90.0% of label claims would be caused by the analytical error. Based on generally agreed specifications for compounded preparations, this preparation could be considered stable under the tested conditions as its initial assay was acceptable (90.0% to 110.0% of label claim) [22–23] and recovery at all time points was not less than 90.0% of the initial concentration [14–21].

Proglycem 50 mg/mL, the oral suspension of diazoxide available in the USA, contains the following ingredients: chocolate mint flavor, alcohol, sorbitol, chocolate cream flavor, propylene glycol, magnesium aluminum silicate, carboxymethycellulose sodium, mint flavor, sodium benzoate, methylparaben, poloxamer 188, propylparaben, and purified water. Hydrochloric acid or sodium hydroxide may be added to adjust pH. It is intended to be stored at controlled room temperature [12]. Suspensions evaluated in the current study were prepared using Oral Mix and Oral Mix SF. Oral Mix contains: purified water, sucrose, glycerin, sorbitol, flavoring, microcrystalline cellulose, carboxymethylcellulose sodium, xantham gum and carrageenan. It is buffered with sodium citrate and citric acid and preserved with potassium sorbate and methylparaben. Oral Mix SF contains similar ingredients the notable difference that sucrose has been replaced by sodium saccharin. From a functional perspective, these three suspensions are similar in nature. As the commercial suspension was known to be stable at room temperature, it is not surprising that we observed at least three months of stability with the compounded preparations. Also, the commercial suspension is chocolate-mint flavored. This is an unusual flavour for a pharmaceutical preparation, suggesting a very bitter taste for diazoxide. As the commercial suspension is not available in Canada, it was not possible to evaluate its organoleptic properties. However, the compounded preparations were uniform white suspensions having a neutral smell and a very bitter taste. A concentration of 10 mg/mL rather than 50 mg/mL slightly reduced the bitterness and will provide manageable volumes of administration for newborns and infants.

This study evaluated the effect of environmental factors on the stability of diazoxide suspensions (10 mg/mL) prepared with Oral Mix and Oral Mix SF. Preparations were compounded using bulk powder and capsules and stored in amber plastic oral syringes and amber PET bottles at 5°C and 25°C for up to 90 days. None of these variables had a significant impact on the stability of the suspensions. Sedimentation of the suspensions was observed after only 7 days. However, shaking by hand was sufficient to reconstitute the products to acceptable suspensions.

To our knowledge, this is the first publication reporting the stability of compounded preparations of diazoxide using a stability-indicating HPLC method. It will provide compounding pharmacist with the required data to use diazoxide in the pediatric population and assign safe beyond-use dates based on sound data.

## Conclusion

A stability-indicating HPLC-UV method was developed to evaluate the concentration of diazoxide in oral suspensions. A stability study was conducted using compounded suspensions of this drug. Diazoxide 10 mg/mL prepared from bulk powder or capsules using Oral Mix or Oral Mix Sugar Free and stored in amber plastic oral syringes or amber PET bottles remained chemically stable for at least 90 days at both 5°C and 25°C.

## Supporting Information

S1 Appendix HPLC results. Excel file containing all reported HPLC results.
(XLSX)

S2 Appendix Browsable stability results. Archive containing the HPLC stability results as browsable html pages.
(ZIP)
