# Supplementary material for: Stability of Diazoxide in Extemporaneously Compounded Oral Suspensions
Source: PLoS One. 2016 Oct 11;11(10):e0164577. doi: 10.1371/journal.pone.0164577 (PMC5058506; doi:10.1371/journal.pone.0164577)
Supplement: S2 Appendix — Archive containing the HPLC stability results as browsable html pages. (ZIP) [file pone.0164577.s002.zip › diazoxide_html_results/diazoxide_syringe.html]

Stability Study Cruncher


### Preparation: bulk-oralmix, Lot: a, Condition: syringe-5

| Days | Assay (mg/mL) | | | % of initial | | | n |  |
| --- | --- | --- | --- | --- | --- | --- | --- | --- |
| 0 | 9.25 | ± | 0.25 |  |  |  | 3 | time zero |
| 7 | 8.90 | ± | 0.44 | 96.2 | ± | 4.8 | 3 | time point |
| 14 | 9.61 | ± | 0.30 | 103.9 | ± | 3.2 | 3 | time point |
| 30 | 8.87 | ± | 0.43 | 95.8 | ± | 4.7 | 3 | time point |
| 45 | 9.70 | ± | 0.35 | 104.9 | ± | 3.8 | 3 | time point |
| 60 | 8.87 | ± | 0.51 | 95.8 | ± | 5.5 | 3 | time point |
| 75 | 9.47 | ± | 0.28 | 102.3 | ± | 3.0 | 3 | time point |
| 90 | 9.43 | ± | 0.43 | 101.9 | ± | 4.6 | 3 | time point |

### Preparation: tablet-oralmix, Lot: a, Condition: syringe-5

| Days | Assay (mg/mL) | | | % of initial | | | n |  |
| --- | --- | --- | --- | --- | --- | --- | --- | --- |
| 0 | 10.01 | ± | 0.14 |  |  |  | 3 | time zero |
| 7 | 10.24 | ± | 0.51 | 102.3 | ± | 5.1 | 3 | time point |
| 14 | 10.39 | ± | 0.11 | 103.8 | ± | 1.1 | 3 | time point |
| 30 | 9.94 | ± | 0.26 | 99.3 | ± | 2.5 | 3 | time point |
| 45 | 9.97 | ± | 0.20 | 99.6 | ± | 2.0 | 3 | time point |
| 60 | 9.84 | ± | 0.23 | 98.3 | ± | 2.3 | 3 | time point |
| 75 | 10.28 | ± | 0.40 | 102.7 | ± | 4.0 | 3 | time point |
| 90 | 9.59 | ± | 0.05 | 95.8 | ± | 0.5 | 3 | time point |

### Preparation: bulk-oralmix, Lot: a, Condition: syringe-25

| Days | Assay (mg/mL) | | | % of initial | | | n |  |
| --- | --- | --- | --- | --- | --- | --- | --- | --- |
| 0 | 9.25 | ± | 0.25 |  |  |  | 3 | time zero |
| 7 | 8.77 | ± | 0.20 | 94.8 | ± | 2.2 | 3 | time point |
| 14 | 9.29 | ± | 0.27 | 100.4 | ± | 2.9 | 3 | time point |
| 30 | 9.30 | ± | 0.47 | 100.5 | ± | 5.1 | 3 | time point |
| 45 | 8.80 | ± | 0.32 | 95.1 | ± | 3.4 | 3 | time point |
| 60 | 9.10 | ± | 0.19 | 98.3 | ± | 2.0 | 3 | time point |
| 75 | 9.41 | ± | 0.29 | 101.7 | ± | 3.1 | 3 | time point |
| 90 | 9.19 | ± | 0.16 | 99.3 | ± | 1.8 | 3 | time point |

### Preparation: tablet-oralmix, Lot: a, Condition: syringe-25

| Days | Assay (mg/mL) | | | % of initial | | | n |  |
| --- | --- | --- | --- | --- | --- | --- | --- | --- |
| 0 | 10.01 | ± | 0.14 |  |  |  | 3 | time zero |
| 7 | 9.75 | ± | 0.23 | 97.4 | ± | 2.3 | 3 | time point |
| 14 | 10.25 | ± | 0.34 | 102.3 | ± | 3.4 | 3 | time point |
| 30 | 10.02 | ± | 0.07 | 100.1 | ± | 0.7 | 3 | time point |
| 45 | 9.80 | ± | 0.07 | 97.9 | ± | 0.7 | 3 | time point |
| 60 | 10.01 | ± | 0.13 | 100.0 | ± | 1.3 | 3 | time point |
| 75 | 10.05 | ± | 0.13 | 100.4 | ± | 1.3 | 3 | time point |
| 90 | 9.56 | ± | 0.13 | 95.5 | ± | 1.3 | 3 | time point |

### Preparation: bulk-oralmixsf, Lot: a, Condition: syringe-5

| Days | Assay (mg/mL) | | | % of initial | | | n |  |
| --- | --- | --- | --- | --- | --- | --- | --- | --- |
| 0 | 9.98 | ± | 0.31 |  |  |  | 3 | time zero |
| 7 | 9.59 | ± | 0.56 | 96.2 | ± | 5.6 | 3 | time point |
| 14 | 9.32 | ± | 0.39 | 93.5 | ± | 3.9 | 3 | time point |
| 30 | 9.51 | ± | 0.24 | 95.3 | ± | 2.4 | 3 | time point |
| 45 | 9.49 | ± | 0.27 | 95.1 | ± | 2.7 | 3 | time point |
| 60 | 9.68 | ± | 0.20 | 97.1 | ± | 2.0 | 3 | time point |
| 75 | 9.71 | ± | 0.27 | 97.4 | ± | 2.7 | 3 | time point |
| 90 | 9.68 | ± | 0.48 | 97.1 | ± | 4.8 | 3 | time point |

### Preparation: tablet-oralmixsf, Lot: a, Condition: syringe-5

| Days | Assay (mg/mL) | | | % of initial | | | n |  |
| --- | --- | --- | --- | --- | --- | --- | --- | --- |
| 0 | 10.05 | ± | 0.08 |  |  |  | 3 | time zero |
| 7 | 9.63 | ± | 0.44 | 95.9 | ± | 4.4 | 3 | time point |
| 14 | 9.96 | ± | 0.48 | 99.1 | ± | 4.8 | 3 | time point |
| 30 | 9.80 | ± | 0.29 | 97.5 | ± | 2.9 | 3 | time point |
| 45 | 9.85 | ± | 0.30 | 98.0 | ± | 2.9 | 3 | time point |
| 60 | 10.20 | ± | 0.32 | 101.5 | ± | 3.1 | 3 | time point |
| 75 | 10.21 | ± | 0.49 | 101.7 | ± | 4.9 | 3 | time point |
| 90 | 10.23 | ± | 0.57 | 101.9 | ± | 5.7 | 3 | time point |

### Preparation: bulk-oralmixsf, Lot: a, Condition: syringe-25

| Days | Assay (mg/mL) | | | % of initial | | | n |  |
| --- | --- | --- | --- | --- | --- | --- | --- | --- |
| 0 | 9.98 | ± | 0.31 |  |  |  | 3 | time zero |
| 7 | 9.18 | ± | 0.14 | 92.0 | ± | 1.4 | 3 | time point |
| 14 | 9.56 | ± | 0.97 | 95.9 | ± | 9.7 | 3 | time point |
| 30 | 9.43 | ± | 0.23 | 94.5 | ± | 2.3 | 3 | time point |
| 45 | 9.60 | ± | 0.37 | 96.3 | ± | 3.7 | 3 | time point |
| 60 | 9.27 | ± | 0.22 | 93.0 | ± | 2.3 | 3 | time point |
| 75 | 9.36 | ± | 0.11 | 93.8 | ± | 1.1 | 3 | time point |
| 90 | 9.53 | ± | 0.26 | 95.6 | ± | 2.6 | 3 | time point |

### Preparation: tablet-oralmixsf, Lot: a, Condition: syringe-25

| Days | Assay (mg/mL) | | | % of initial | | | n |  |
| --- | --- | --- | --- | --- | --- | --- | --- | --- |
| 0 | 10.05 | ± | 0.08 |  |  |  | 3 | time zero |
| 7 | 9.71 | ± | 0.20 | 96.6 | ± | 2.0 | 3 | time point |
| 14 | 9.21 | ± | 0.12 | 91.7 | ± | 1.2 | 3 | time point |
| 30 | 9.83 | ± | 0.26 | 97.9 | ± | 2.6 | 3 | time point |
| 45 | 9.82 | ± | 0.37 | 97.8 | ± | 3.7 | 3 | time point |
| 60 | 10.06 | ± | 0.58 | 100.1 | ± | 5.8 | 3 | time point |
| 75 | 10.12 | ± | 0.07 | 100.7 | ± | 0.7 | 3 | time point |
| 90 | 10.28 | ± | 0.32 | 102.3 | ± | 3.2 | 3 | time point |
