# Supplementary material for: Stability of Diazoxide in Extemporaneously Compounded Oral Suspensions
Source: PLoS One. 2016 Oct 11;11(10):e0164577. doi: 10.1371/journal.pone.0164577 (PMC5058506; doi:10.1371/journal.pone.0164577)
Supplement: S2 Appendix — Archive containing the HPLC stability results as browsable html pages. (ZIP) [file pone.0164577.s002.zip › diazoxide_html_results/diazoxide_syringe/index.html?preparation=bulk-oralmix&lot=a.html]

Stability Study Cruncher


### Preparation: bulk-oralmix, Lot: a

Assay: 9.25 ± 0.25 mg/mL (n = 3).

| Input String | Area | Cal Id | Cal Slope | Assay |  |
| --- | --- | --- | --- | --- | --- |
| diazoxide\_bulk-oralmix\_a;3207849;;calt0om210;time zero | 3207849 | calt0om210 | 356227 | 9.01 | calibration |
| diazoxide\_bulk-oralmix\_a;3458561;;calt0om200;time zero | 3458561 | calt0om200 | 374038 | 9.25 | calibration |
| diazoxide\_bulk-oralmix\_a;3556179;;calt0om200;time zero | 3556179 | calt0om200 | 374038 | 9.51 | calibration |
