# Supplementary material for: Stability of Diazoxide in Extemporaneously Compounded Oral Suspensions
Source: PLoS One. 2016 Oct 11;11(10):e0164577. doi: 10.1371/journal.pone.0164577 (PMC5058506; doi:10.1371/journal.pone.0164577)
Supplement: S2 Appendix — Archive containing the HPLC stability results as browsable html pages. (ZIP) [file pone.0164577.s002.zip › diazoxide_html_results/diazoxide_syringe/index.html?preparation=bulk-oralmix&lot=a&condition=syringe-5&time=7.html]

Stability Study Cruncher


### Preparation: bulk-oralmix, Lot: a, Condition: syringe-5, Time: 7

Assay (mg/mL): 8.90 ± 0.44 (n = 3);
Assay (%TZ): 96.2 ± 4.8 (n = 3).

| Input String | Area | Cal Id | Cal Slope | Assay | Assay TZ | Assay %TZ |  |
| --- | --- | --- | --- | --- | --- | --- | --- |
| diazoxide\_bulk-oralmix\_a\_syringe-5\_7;3144747;;cal7om200;stability | 3144747 | cal7om200 | 373935 | 8.41 | 9.25 | 90.9 | calibration, time zero |
| diazoxide\_bulk-oralmix\_a\_syringe-5\_7;3467887;;cal7om200;stability | 3467887 | cal7om200 | 373935 | 9.27 | 9.25 | 100.2 | calibration, time zero |
| diazoxide\_bulk-oralmix\_a\_syringe-5\_7;3368907;;cal7om200;stability | 3368907 | cal7om200 | 373935 | 9.01 | 9.25 | 97.4 | calibration, time zero |
