# Supplementary material for: Stability of Diazoxide in Extemporaneously Compounded Oral Suspensions
Source: PLoS One. 2016 Oct 11;11(10):e0164577. doi: 10.1371/journal.pone.0164577 (PMC5058506; doi:10.1371/journal.pone.0164577)
Supplement: S2 Appendix — Archive containing the HPLC stability results as browsable html pages. (ZIP) [file pone.0164577.s002.zip › diazoxide_html_results/diazoxide_syringe/index.html?preparation=bulk-oralmix&lot=a&condition=syringe-5&time=30.html]

Stability Study Cruncher


### Preparation: bulk-oralmix, Lot: a, Condition: syringe-5, Time: 30

Assay (mg/mL): 8.87 ± 0.43 (n = 3);
Assay (%TZ): 95.8 ± 4.7 (n = 3).

| Input String | Area | Cal Id | Cal Slope | Assay | Assay TZ | Assay %TZ |  |
| --- | --- | --- | --- | --- | --- | --- | --- |
| diazoxide\_bulk-oralmix\_a\_syringe-5\_30;2999710;;cal14om210;stability | 2999710 | cal14om210 | 358223 | 8.37 | 9.25 | 90.5 | calibration, time zero |
| diazoxide\_bulk-oralmix\_a\_syringe-5\_30;3242053;;cal14om210;stability | 3242053 | cal14om210 | 358223 | 9.05 | 9.25 | 97.8 | calibration, time zero |
| diazoxide\_bulk-oralmix\_a\_syringe-5\_30;3286268;;cal14om210;stability | 3286268 | cal14om210 | 358223 | 9.17 | 9.25 | 99.1 | calibration, time zero |
