# Supplementary material for: Stability of Diazoxide in Extemporaneously Compounded Oral Suspensions
Source: PLoS One. 2016 Oct 11;11(10):e0164577. doi: 10.1371/journal.pone.0164577 (PMC5058506; doi:10.1371/journal.pone.0164577)
Supplement: S2 Appendix — Archive containing the HPLC stability results as browsable html pages. (ZIP) [file pone.0164577.s002.zip › diazoxide_html_results/diazoxide_syringe/index.html?preparation=bulk-oralmix&lot=a&condition=syringe-5&time=60.html]

Stability Study Cruncher


### Preparation: bulk-oralmix, Lot: a, Condition: syringe-5, Time: 60

Assay (mg/mL): 8.87 ± 0.51 (n = 3);
Assay (%TZ): 95.8 ± 5.5 (n = 3).

| Input String | Area | Cal Id | Cal Slope | Assay | Assay TZ | Assay %TZ |  |
| --- | --- | --- | --- | --- | --- | --- | --- |
| diazoxide\_bulk-oralmix\_a\_syringe-5\_60;2970054;;cal14om210;stability | 2970054 | cal14om210 | 358223 | 8.29 | 9.25 | 89.6 | calibration, time zero |
| diazoxide\_bulk-oralmix\_a\_syringe-5\_60;3238157;;cal14om210;stability | 3238157 | cal14om210 | 358223 | 9.04 | 9.25 | 97.7 | calibration, time zero |
| diazoxide\_bulk-oralmix\_a\_syringe-5\_60;3321964;;cal14om210;stability | 3321964 | cal14om210 | 358223 | 9.27 | 9.25 | 100.2 | calibration, time zero |
