# Supplementary material for: Stability of Diazoxide in Extemporaneously Compounded Oral Suspensions
Source: PLoS One. 2016 Oct 11;11(10):e0164577. doi: 10.1371/journal.pone.0164577 (PMC5058506; doi:10.1371/journal.pone.0164577)
Supplement: S2 Appendix — Archive containing the HPLC stability results as browsable html pages. (ZIP) [file pone.0164577.s002.zip › diazoxide_html_results/diazoxide_syringe/index.html?preparation=bulk-oralmix&lot=a&condition=syringe-5&time=90.html]

Stability Study Cruncher


### Preparation: bulk-oralmix, Lot: a, Condition: syringe-5, Time: 90

Assay (mg/mL): 9.43 ± 0.43 (n = 3);
Assay (%TZ): 101.9 ± 4.6 (n = 3).

| Input String | Area | Cal Id | Cal Slope | Assay | Assay TZ | Assay %TZ |  |
| --- | --- | --- | --- | --- | --- | --- | --- |
| diazoxide\_bulk-oralmix\_a\_syringe-5\_90;3322253;;cal75om210;stability | 3322253 | cal75om210 | 358017 | 9.28 | 9.25 | 100.3 | calibration, time zero |
| diazoxide\_bulk-oralmix\_a\_syringe-5\_90;3257142;;cal75om210;stability | 3257142 | cal75om210 | 358017 | 9.10 | 9.25 | 98.3 | calibration, time zero |
| diazoxide\_bulk-oralmix\_a\_syringe-5\_90;3549908;;cal75om210;stability | 3549908 | cal75om210 | 358017 | 9.92 | 9.25 | 107.2 | calibration, time zero |
