# Supplementary material for: Stability of Diazoxide in Extemporaneously Compounded Oral Suspensions
Source: PLoS One. 2016 Oct 11;11(10):e0164577. doi: 10.1371/journal.pone.0164577 (PMC5058506; doi:10.1371/journal.pone.0164577)
Supplement: S2 Appendix — Archive containing the HPLC stability results as browsable html pages. (ZIP) [file pone.0164577.s002.zip › diazoxide_html_results/diazoxide_syringe/index.html?preparation=tablet-oralmix&lot=a&condition=syringe-5&time=7.html]

Stability Study Cruncher


### Preparation: tablet-oralmix, Lot: a, Condition: syringe-5, Time: 7

Assay (mg/mL): 10.24 ± 0.51 (n = 3);
Assay (%TZ): 102.3 ± 5.1 (n = 3).

| Input String | Area | Cal Id | Cal Slope | Assay | Assay TZ | Assay %TZ |  |
| --- | --- | --- | --- | --- | --- | --- | --- |
| diazoxide\_tablet-oralmix\_a\_syringe-5\_7;4039638;;cal7om200;stability | 4039638 | cal7om200 | 373935 | 10.80 | 10.01 | 107.9 | calibration, time zero |
| diazoxide\_tablet-oralmix\_a\_syringe-5\_7;3674726;;cal7om200;stability | 3674726 | cal7om200 | 373935 | 9.83 | 10.01 | 98.2 | calibration, time zero |
| diazoxide\_tablet-oralmix\_a\_syringe-5\_7;3770863;;cal7om200;stability | 3770863 | cal7om200 | 373935 | 10.08 | 10.01 | 100.7 | calibration, time zero |
