# Supplementary material for: Stability of Diazoxide in Extemporaneously Compounded Oral Suspensions
Source: PLoS One. 2016 Oct 11;11(10):e0164577. doi: 10.1371/journal.pone.0164577 (PMC5058506; doi:10.1371/journal.pone.0164577)
Supplement: S2 Appendix — Archive containing the HPLC stability results as browsable html pages. (ZIP) [file pone.0164577.s002.zip › diazoxide_html_results/diazoxide_syringe/index.html?preparation=tablet-oralmix&lot=a&condition=syringe-5&time=60.html]

Stability Study Cruncher


### Preparation: tablet-oralmix, Lot: a, Condition: syringe-5, Time: 60

Assay (mg/mL): 9.84 ± 0.23 (n = 3);
Assay (%TZ): 98.3 ± 2.3 (n = 3).

| Input String | Area | Cal Id | Cal Slope | Assay | Assay TZ | Assay %TZ |  |
| --- | --- | --- | --- | --- | --- | --- | --- |
| diazoxide\_tablet-oralmix\_a\_syringe-5\_60;3468811;;cal14om210;stability | 3468811 | cal14om210 | 358223 | 9.68 | 10.01 | 96.7 | calibration, time zero |
| diazoxide\_tablet-oralmix\_a\_syringe-5\_60;3619361;;cal14om210;stability | 3619361 | cal14om210 | 358223 | 10.10 | 10.01 | 100.9 | calibration, time zero |
| diazoxide\_tablet-oralmix\_a\_syringe-5\_60;3491580;;cal14om210;stability | 3491580 | cal14om210 | 358223 | 9.75 | 10.01 | 97.4 | calibration, time zero |
