# Supplementary material for: Stability of Diazoxide in Extemporaneously Compounded Oral Suspensions
Source: PLoS One. 2016 Oct 11;11(10):e0164577. doi: 10.1371/journal.pone.0164577 (PMC5058506; doi:10.1371/journal.pone.0164577)
Supplement: S2 Appendix — Archive containing the HPLC stability results as browsable html pages. (ZIP) [file pone.0164577.s002.zip › diazoxide_html_results/diazoxide_syringe/index.html?preparation=tablet-oralmix&lot=a&condition=syringe-5&time=75.html]

Stability Study Cruncher


### Preparation: tablet-oralmix, Lot: a, Condition: syringe-5, Time: 75

Assay (mg/mL): 10.28 ± 0.40 (n = 3);
Assay (%TZ): 102.7 ± 4.0 (n = 3).

| Input String | Area | Cal Id | Cal Slope | Assay | Assay TZ | Assay %TZ |  |
| --- | --- | --- | --- | --- | --- | --- | --- |
| diazoxide\_tablet-oralmix\_a\_syringe-5\_75;3566401;;cal75om210;stability | 3566401 | cal75om210 | 358017 | 9.96 | 10.01 | 99.5 | calibration, time zero |
| diazoxide\_tablet-oralmix\_a\_syringe-5\_75;3631990;;cal75om210;stability | 3631990 | cal75om210 | 358017 | 10.14 | 10.01 | 101.3 | calibration, time zero |
| diazoxide\_tablet-oralmix\_a\_syringe-5\_75;3839670;;cal75om210;stability | 3839670 | cal75om210 | 358017 | 10.72 | 10.01 | 107.1 | calibration, time zero |
