# Supplementary material for: Stability of Diazoxide in Extemporaneously Compounded Oral Suspensions
Source: PLoS One. 2016 Oct 11;11(10):e0164577. doi: 10.1371/journal.pone.0164577 (PMC5058506; doi:10.1371/journal.pone.0164577)
Supplement: S2 Appendix — Archive containing the HPLC stability results as browsable html pages. (ZIP) [file pone.0164577.s002.zip › diazoxide_html_results/diazoxide_syringe/index.html?preparation=bulk-oralmix&lot=a&condition=syringe-25&time=7.html]

Stability Study Cruncher


### Preparation: bulk-oralmix, Lot: a, Condition: syringe-25, Time: 7

Assay (mg/mL): 8.77 ± 0.20 (n = 3);
Assay (%TZ): 94.8 ± 2.2 (n = 3).

| Input String | Area | Cal Id | Cal Slope | Assay | Assay TZ | Assay %TZ |  |
| --- | --- | --- | --- | --- | --- | --- | --- |
| diazoxide\_bulk-oralmix\_a\_syringe-25\_7;3201035;;cal7om200;stability | 3201035 | cal7om200 | 373935 | 8.56 | 9.25 | 92.5 | calibration, time zero |
| diazoxide\_bulk-oralmix\_a\_syringe-25\_7;3349976;;cal7om200;stability | 3349976 | cal7om200 | 373935 | 8.96 | 9.25 | 96.8 | calibration, time zero |
| diazoxide\_bulk-oralmix\_a\_syringe-25\_7;3289009;;cal7om200;stability | 3289009 | cal7om200 | 373935 | 8.80 | 9.25 | 95.1 | calibration, time zero |
