# Supplementary material for: Stability of Diazoxide in Extemporaneously Compounded Oral Suspensions
Source: PLoS One. 2016 Oct 11;11(10):e0164577. doi: 10.1371/journal.pone.0164577 (PMC5058506; doi:10.1371/journal.pone.0164577)
Supplement: S2 Appendix — Archive containing the HPLC stability results as browsable html pages. (ZIP) [file pone.0164577.s002.zip › diazoxide_html_results/diazoxide_syringe/index.html?preparation=tablet-oralmixsf&lot=a.html]

Stability Study Cruncher


### Preparation: tablet-oralmixsf, Lot: a

Assay: 10.05 ± 0.08 mg/mL (n = 3).

| Input String | Area | Cal Id | Cal Slope | Assay |  |
| --- | --- | --- | --- | --- | --- |
| diazoxide\_tablet-oralmixsf\_a;3785877;;calt0sf200;time zero | 3785877 | calt0sf200 | 374038 | 10.12 | calibration |
| diazoxide\_tablet-oralmixsf\_a;3723665;;calt0sf200;time zero | 3723665 | calt0sf200 | 374038 | 9.96 | calibration |
| diazoxide\_tablet-oralmixsf\_a;3764219;;calt0sf200;time zero | 3764219 | calt0sf200 | 374038 | 10.06 | calibration |
