# Supplementary material for: Stability of Diazoxide in Extemporaneously Compounded Oral Suspensions
Source: PLoS One. 2016 Oct 11;11(10):e0164577. doi: 10.1371/journal.pone.0164577 (PMC5058506; doi:10.1371/journal.pone.0164577)
Supplement: S2 Appendix — Archive containing the HPLC stability results as browsable html pages. (ZIP) [file pone.0164577.s002.zip › diazoxide_html_results/diazoxide_syringe/index.html?preparation=bulk-oralmix&lot=a&condition=syringe-25&time=75.html]

Stability Study Cruncher


### Preparation: bulk-oralmix, Lot: a, Condition: syringe-25, Time: 75

Assay (mg/mL): 9.41 ± 0.29 (n = 3);
Assay (%TZ): 101.7 ± 3.1 (n = 3).

| Input String | Area | Cal Id | Cal Slope | Assay | Assay TZ | Assay %TZ |  |
| --- | --- | --- | --- | --- | --- | --- | --- |
| diazoxide\_bulk-oralmix\_a\_syringe-25\_75;3311608;;cal75om210;stability | 3311608 | cal75om210 | 358017 | 9.25 | 9.25 | 100.0 | calibration, time zero |
| diazoxide\_bulk-oralmix\_a\_syringe-25\_75;3488678;;cal75om210;stability | 3488678 | cal75om210 | 358017 | 9.74 | 9.25 | 105.3 | calibration, time zero |
| diazoxide\_bulk-oralmix\_a\_syringe-25\_75;3304981;;cal75om210;stability | 3304981 | cal75om210 | 358017 | 9.23 | 9.25 | 99.8 | calibration, time zero |
