# Supplementary material for: Stability of Diazoxide in Extemporaneously Compounded Oral Suspensions
Source: PLoS One. 2016 Oct 11;11(10):e0164577. doi: 10.1371/journal.pone.0164577 (PMC5058506; doi:10.1371/journal.pone.0164577)
Supplement: S2 Appendix — Archive containing the HPLC stability results as browsable html pages. (ZIP) [file pone.0164577.s002.zip › diazoxide_html_results/diazoxide_syringe/index.html?preparation=bulk-oralmix&lot=a&condition=syringe-25&time=90.html]

Stability Study Cruncher


### Preparation: bulk-oralmix, Lot: a, Condition: syringe-25, Time: 90

Assay (mg/mL): 9.19 ± 0.16 (n = 3);
Assay (%TZ): 99.3 ± 1.8 (n = 3).

| Input String | Area | Cal Id | Cal Slope | Assay | Assay TZ | Assay %TZ |  |
| --- | --- | --- | --- | --- | --- | --- | --- |
| diazoxide\_bulk-oralmix\_a\_syringe-25\_90;3247492;;cal75om210;stability | 3247492 | cal75om210 | 358017 | 9.07 | 9.25 | 98.0 | calibration, time zero |
| diazoxide\_bulk-oralmix\_a\_syringe-25\_90;3357368;;cal75om210;stability | 3357368 | cal75om210 | 358017 | 9.38 | 9.25 | 101.3 | calibration, time zero |
| diazoxide\_bulk-oralmix\_a\_syringe-25\_90;3268079;;cal75om210;stability | 3268079 | cal75om210 | 358017 | 9.13 | 9.25 | 98.7 | calibration, time zero |
