# Supplementary material for: Stability of Diazoxide in Extemporaneously Compounded Oral Suspensions
Source: PLoS One. 2016 Oct 11;11(10):e0164577. doi: 10.1371/journal.pone.0164577 (PMC5058506; doi:10.1371/journal.pone.0164577)
Supplement: S2 Appendix — Archive containing the HPLC stability results as browsable html pages. (ZIP) [file pone.0164577.s002.zip › diazoxide_html_results/diazoxide_syringe/index.html?preparation=tablet-oralmix&lot=a&condition=syringe-25&time=7.html]

Stability Study Cruncher


### Preparation: tablet-oralmix, Lot: a, Condition: syringe-25, Time: 7

Assay (mg/mL): 9.75 ± 0.23 (n = 3);
Assay (%TZ): 97.4 ± 2.3 (n = 3).

| Input String | Area | Cal Id | Cal Slope | Assay | Assay TZ | Assay %TZ |  |
| --- | --- | --- | --- | --- | --- | --- | --- |
| diazoxide\_tablet-oralmix\_a\_syringe-25\_7;3673919;;cal7om200;stability | 3673919 | cal7om200 | 373935 | 9.83 | 10.01 | 98.2 | calibration, time zero |
| diazoxide\_tablet-oralmix\_a\_syringe-25\_7;3718569;;cal7om200;stability | 3718569 | cal7om200 | 373935 | 9.94 | 10.01 | 99.3 | calibration, time zero |
| diazoxide\_tablet-oralmix\_a\_syringe-25\_7;3549485;;cal7om200;stability | 3549485 | cal7om200 | 373935 | 9.49 | 10.01 | 94.8 | calibration, time zero |
