# Supplementary material for: Stability of Diazoxide in Extemporaneously Compounded Oral Suspensions
Source: PLoS One. 2016 Oct 11;11(10):e0164577. doi: 10.1371/journal.pone.0164577 (PMC5058506; doi:10.1371/journal.pone.0164577)
Supplement: S2 Appendix — Archive containing the HPLC stability results as browsable html pages. (ZIP) [file pone.0164577.s002.zip › diazoxide_html_results/diazoxide_syringe/index.html?preparation=tablet-oralmix&lot=a&condition=syringe-25&time=60.html]

Stability Study Cruncher


### Preparation: tablet-oralmix, Lot: a, Condition: syringe-25, Time: 60

Assay (mg/mL): 10.01 ± 0.13 (n = 3);
Assay (%TZ): 100.0 ± 1.3 (n = 3).

| Input String | Area | Cal Id | Cal Slope | Assay | Assay TZ | Assay %TZ |  |
| --- | --- | --- | --- | --- | --- | --- | --- |
| diazoxide\_tablet-oralmix\_a\_syringe-25\_60;3560099;;cal14om210;stability | 3560099 | cal14om210 | 358223 | 9.94 | 10.01 | 99.3 | calibration, time zero |
| diazoxide\_tablet-oralmix\_a\_syringe-25\_60;3639457;;cal14om210;stability | 3639457 | cal14om210 | 358223 | 10.16 | 10.01 | 101.5 | calibration, time zero |
| diazoxide\_tablet-oralmix\_a\_syringe-25\_60;3552785;;cal14om210;stability | 3552785 | cal14om210 | 358223 | 9.92 | 10.01 | 99.1 | calibration, time zero |
