# Supplementary material for: Stability of Diazoxide in Extemporaneously Compounded Oral Suspensions
Source: PLoS One. 2016 Oct 11;11(10):e0164577. doi: 10.1371/journal.pone.0164577 (PMC5058506; doi:10.1371/journal.pone.0164577)
Supplement: S2 Appendix — Archive containing the HPLC stability results as browsable html pages. (ZIP) [file pone.0164577.s002.zip › diazoxide_html_results/diazoxide_syringe/index.html?preparation=bulk-oralmixsf&lot=a.html]

Stability Study Cruncher


### Preparation: bulk-oralmixsf, Lot: a

Assay: 9.98 ± 0.31 mg/mL (n = 3).

| Input String | Area | Cal Id | Cal Slope | Assay |  |
| --- | --- | --- | --- | --- | --- |
| diazoxide\_bulk-oralmixsf\_a;3847949;;calt0sf200;time zero | 3847949 | calt0sf200 | 374038 | 10.29 | calibration |
| diazoxide\_bulk-oralmixsf\_a;3619278;;calt0sf200;time zero | 3619278 | calt0sf200 | 374038 | 9.68 | calibration |
| diazoxide\_bulk-oralmixsf\_a;3725934;;calt0sf200;time zero | 3725934 | calt0sf200 | 374038 | 9.96 | calibration |
