# Supplementary material for: Stability of Diazoxide in Extemporaneously Compounded Oral Suspensions
Source: PLoS One. 2016 Oct 11;11(10):e0164577. doi: 10.1371/journal.pone.0164577 (PMC5058506; doi:10.1371/journal.pone.0164577)
Supplement: S2 Appendix — Archive containing the HPLC stability results as browsable html pages. (ZIP) [file pone.0164577.s002.zip › diazoxide_html_results/diazoxide_syringe/index.html?preparation=bulk-oralmixsf&lot=a&condition=syringe-5&time=7.html]

Stability Study Cruncher


### Preparation: bulk-oralmixsf, Lot: a, Condition: syringe-5, Time: 7

Assay (mg/mL): 9.59 ± 0.56 (n = 3);
Assay (%TZ): 96.2 ± 5.6 (n = 3).

| Input String | Area | Cal Id | Cal Slope | Assay | Assay TZ | Assay %TZ |  |
| --- | --- | --- | --- | --- | --- | --- | --- |
| diazoxide\_bulk-oralmixsf\_a\_syringe-5\_7;3259263;;cal7sf210;stability | 3259263 | cal7sf210 | 355486 | 9.17 | 9.98 | 91.9 | calibration, time zero |
| diazoxide\_bulk-oralmixsf\_a\_syringe-5\_7;3505350;;cal7sf200;stability | 3505350 | cal7sf200 | 373260 | 9.39 | 9.98 | 94.1 | calibration, time zero |
| diazoxide\_bulk-oralmixsf\_a\_syringe-5\_7;3816226;;cal7sf200;stability | 3816226 | cal7sf200 | 373260 | 10.22 | 9.98 | 102.5 | calibration, time zero |
