# Supplementary material for: Stability of Diazoxide in Extemporaneously Compounded Oral Suspensions
Source: PLoS One. 2016 Oct 11;11(10):e0164577. doi: 10.1371/journal.pone.0164577 (PMC5058506; doi:10.1371/journal.pone.0164577)
Supplement: S2 Appendix — Archive containing the HPLC stability results as browsable html pages. (ZIP) [file pone.0164577.s002.zip › diazoxide_html_results/diazoxide_syringe/index.html?preparation=bulk-oralmixsf&lot=a&condition=syringe-5&time=14.html]

Stability Study Cruncher


### Preparation: bulk-oralmixsf, Lot: a, Condition: syringe-5, Time: 14

Assay (mg/mL): 9.32 ± 0.39 (n = 3);
Assay (%TZ): 93.5 ± 3.9 (n = 3).

| Input String | Area | Cal Id | Cal Slope | Assay | Assay TZ | Assay %TZ |  |
| --- | --- | --- | --- | --- | --- | --- | --- |
| diazoxide\_bulk-oralmixsf\_a\_syringe-5\_14;3313443;;cal14sf210;stability | 3313443 | cal14sf210 | 359483 | 9.22 | 9.98 | 92.4 | calibration, time zero |
| diazoxide\_bulk-oralmixsf\_a\_syringe-5\_14;3232798;;cal14sf210;stability | 3232798 | cal14sf210 | 359483 | 8.99 | 9.98 | 90.2 | calibration, time zero |
| diazoxide\_bulk-oralmixsf\_a\_syringe-5\_14;3507448;;cal14sf210;stability | 3507448 | cal14sf210 | 359483 | 9.76 | 9.98 | 97.8 | calibration, time zero |
