# Supplementary material for: Stability of Diazoxide in Extemporaneously Compounded Oral Suspensions
Source: PLoS One. 2016 Oct 11;11(10):e0164577. doi: 10.1371/journal.pone.0164577 (PMC5058506; doi:10.1371/journal.pone.0164577)
Supplement: S2 Appendix — Archive containing the HPLC stability results as browsable html pages. (ZIP) [file pone.0164577.s002.zip › diazoxide_html_results/diazoxide_syringe/index.html?preparation=bulk-oralmixsf&lot=a&condition=syringe-5&time=30.html]

Stability Study Cruncher


### Preparation: bulk-oralmixsf, Lot: a, Condition: syringe-5, Time: 30

Assay (mg/mL): 9.51 ± 0.24 (n = 3);
Assay (%TZ): 95.3 ± 2.4 (n = 3).

| Input String | Area | Cal Id | Cal Slope | Assay | Assay TZ | Assay %TZ |  |
| --- | --- | --- | --- | --- | --- | --- | --- |
| diazoxide\_bulk-oralmixsf\_a\_syringe-5\_30;3422329;;cal30sf210;stability | 3422329 | cal30sf210 | 358295 | 9.55 | 9.98 | 95.8 | calibration, time zero |
| diazoxide\_bulk-oralmixsf\_a\_syringe-5\_30;3485522;;cal30sf210;stability | 3485522 | cal30sf210 | 358295 | 9.73 | 9.98 | 97.5 | calibration, time zero |
| diazoxide\_bulk-oralmixsf\_a\_syringe-5\_30;3314039;;cal30sf210;stability | 3314039 | cal30sf210 | 358295 | 9.25 | 9.98 | 92.7 | calibration, time zero |
