# Supplementary material for: Stability of Diazoxide in Extemporaneously Compounded Oral Suspensions
Source: PLoS One. 2016 Oct 11;11(10):e0164577. doi: 10.1371/journal.pone.0164577 (PMC5058506; doi:10.1371/journal.pone.0164577)
Supplement: S2 Appendix — Archive containing the HPLC stability results as browsable html pages. (ZIP) [file pone.0164577.s002.zip › diazoxide_html_results/diazoxide_syringe/index.html?preparation=bulk-oralmixsf&lot=a&condition=syringe-5&time=60.html]

Stability Study Cruncher


### Preparation: bulk-oralmixsf, Lot: a, Condition: syringe-5, Time: 60

Assay (mg/mL): 9.68 ± 0.20 (n = 3);
Assay (%TZ): 97.1 ± 2.0 (n = 3).

| Input String | Area | Cal Id | Cal Slope | Assay | Assay TZ | Assay %TZ |  |
| --- | --- | --- | --- | --- | --- | --- | --- |
| diazoxide\_bulk-oralmixsf\_a\_syringe-5\_60;3404167;;cal60sf210;stability | 3404167 | cal60sf210 | 358176 | 9.50 | 9.98 | 95.3 | calibration, time zero |
| diazoxide\_bulk-oralmixsf\_a\_syringe-5\_60;3455161;;cal60sf210;stability | 3455161 | cal60sf210 | 358176 | 9.65 | 9.98 | 96.7 | calibration, time zero |
| diazoxide\_bulk-oralmixsf\_a\_syringe-5\_60;3543879;;cal60sf210;stability | 3543879 | cal60sf210 | 358176 | 9.89 | 9.98 | 99.2 | calibration, time zero |
