# Supplementary material for: Stability of Diazoxide in Extemporaneously Compounded Oral Suspensions
Source: PLoS One. 2016 Oct 11;11(10):e0164577. doi: 10.1371/journal.pone.0164577 (PMC5058506; doi:10.1371/journal.pone.0164577)
Supplement: S2 Appendix — Archive containing the HPLC stability results as browsable html pages. (ZIP) [file pone.0164577.s002.zip › diazoxide_html_results/diazoxide_syringe/index.html?preparation=tablet-oralmixsf&lot=a&condition=syringe-5&time=14.html]

Stability Study Cruncher


### Preparation: tablet-oralmixsf, Lot: a, Condition: syringe-5, Time: 14

Assay (mg/mL): 9.96 ± 0.48 (n = 3);
Assay (%TZ): 99.1 ± 4.8 (n = 3).

| Input String | Area | Cal Id | Cal Slope | Assay | Assay TZ | Assay %TZ |  |
| --- | --- | --- | --- | --- | --- | --- | --- |
| diazoxide\_tablet-oralmixsf\_a\_syringe-5\_14;3501882;;cal14sf210;stability | 3501882 | cal14sf210 | 359483 | 9.74 | 10.05 | 97.0 | calibration, time zero |
| diazoxide\_tablet-oralmixsf\_a\_syringe-5\_14;3459612;;cal14sf210;stability | 3459612 | cal14sf210 | 359483 | 9.62 | 10.05 | 95.8 | calibration, time zero |
| diazoxide\_tablet-oralmixsf\_a\_syringe-5\_14;3777030;;cal14sf210;stability | 3777030 | cal14sf210 | 359483 | 10.51 | 10.05 | 104.6 | calibration, time zero |
