# Supplementary material for: Stability of Diazoxide in Extemporaneously Compounded Oral Suspensions
Source: PLoS One. 2016 Oct 11;11(10):e0164577. doi: 10.1371/journal.pone.0164577 (PMC5058506; doi:10.1371/journal.pone.0164577)
Supplement: S2 Appendix — Archive containing the HPLC stability results as browsable html pages. (ZIP) [file pone.0164577.s002.zip › diazoxide_html_results/diazoxide_syringe/index.html?preparation=tablet-oralmixsf&lot=a&condition=syringe-5&time=45.html]

Stability Study Cruncher


### Preparation: tablet-oralmixsf, Lot: a, Condition: syringe-5, Time: 45

Assay (mg/mL): 9.85 ± 0.30 (n = 3);
Assay (%TZ): 98.0 ± 2.9 (n = 3).

| Input String | Area | Cal Id | Cal Slope | Assay | Assay TZ | Assay %TZ |  |
| --- | --- | --- | --- | --- | --- | --- | --- |
| diazoxide\_tablet-oralmixsf\_a\_syringe-5\_45;3621139;;cal30sf210;stability | 3621139 | cal30sf210 | 358295 | 10.11 | 10.05 | 100.6 | calibration, time zero |
| diazoxide\_tablet-oralmixsf\_a\_syringe-5\_45;3412901;;cal30sf210;stability | 3412901 | cal30sf210 | 358295 | 9.53 | 10.05 | 94.8 | calibration, time zero |
| diazoxide\_tablet-oralmixsf\_a\_syringe-5\_45;3552906;;cal30sf210;stability | 3552906 | cal30sf210 | 358295 | 9.92 | 10.05 | 98.7 | calibration, time zero |
