# Supplementary material for: Stability of Diazoxide in Extemporaneously Compounded Oral Suspensions
Source: PLoS One. 2016 Oct 11;11(10):e0164577. doi: 10.1371/journal.pone.0164577 (PMC5058506; doi:10.1371/journal.pone.0164577)
Supplement: S2 Appendix — Archive containing the HPLC stability results as browsable html pages. (ZIP) [file pone.0164577.s002.zip › diazoxide_html_results/diazoxide_syringe/index.html?preparation=tablet-oralmixsf&lot=a&condition=syringe-5&time=60.html]

Stability Study Cruncher


### Preparation: tablet-oralmixsf, Lot: a, Condition: syringe-5, Time: 60

Assay (mg/mL): 10.20 ± 0.32 (n = 3);
Assay (%TZ): 101.5 ± 3.1 (n = 3).

| Input String | Area | Cal Id | Cal Slope | Assay | Assay TZ | Assay %TZ |  |
| --- | --- | --- | --- | --- | --- | --- | --- |
| diazoxide\_tablet-oralmixsf\_a\_syringe-5\_60;3591382;;cal60sf210;stability | 3591382 | cal60sf210 | 358176 | 10.03 | 10.05 | 99.8 | calibration, time zero |
| diazoxide\_tablet-oralmixsf\_a\_syringe-5\_60;3783045;;cal60sf210;stability | 3783045 | cal60sf210 | 358176 | 10.56 | 10.05 | 105.1 | calibration, time zero |
| diazoxide\_tablet-oralmixsf\_a\_syringe-5\_60;3583039;;cal60sf210;stability | 3583039 | cal60sf210 | 358176 | 10.00 | 10.05 | 99.6 | calibration, time zero |
