# Supplementary material for: Stability of Diazoxide in Extemporaneously Compounded Oral Suspensions
Source: PLoS One. 2016 Oct 11;11(10):e0164577. doi: 10.1371/journal.pone.0164577 (PMC5058506; doi:10.1371/journal.pone.0164577)
Supplement: S2 Appendix — Archive containing the HPLC stability results as browsable html pages. (ZIP) [file pone.0164577.s002.zip › diazoxide_html_results/diazoxide_syringe/index.html?preparation=tablet-oralmixsf&lot=a&condition=syringe-5&time=75.html]

Stability Study Cruncher


### Preparation: tablet-oralmixsf, Lot: a, Condition: syringe-5, Time: 75

Assay (mg/mL): 10.21 ± 0.49 (n = 3);
Assay (%TZ): 101.7 ± 4.9 (n = 3).

| Input String | Area | Cal Id | Cal Slope | Assay | Assay TZ | Assay %TZ |  |
| --- | --- | --- | --- | --- | --- | --- | --- |
| diazoxide\_tablet-oralmixsf\_a\_syringe-5\_75;3456630;;cal60sf210;stability | 3456630 | cal60sf210 | 358176 | 9.65 | 10.05 | 96.1 | calibration, time zero |
| diazoxide\_tablet-oralmixsf\_a\_syringe-5\_75;3749676;;cal60sf210;stability | 3749676 | cal60sf210 | 358176 | 10.47 | 10.05 | 104.2 | calibration, time zero |
| diazoxide\_tablet-oralmixsf\_a\_syringe-5\_75;3768504;;cal60sf210;stability | 3768504 | cal60sf210 | 358176 | 10.52 | 10.05 | 104.7 | calibration, time zero |
