# Supplementary material for: Stability of Diazoxide in Extemporaneously Compounded Oral Suspensions
Source: PLoS One. 2016 Oct 11;11(10):e0164577. doi: 10.1371/journal.pone.0164577 (PMC5058506; doi:10.1371/journal.pone.0164577)
Supplement: S2 Appendix — Archive containing the HPLC stability results as browsable html pages. (ZIP) [file pone.0164577.s002.zip › diazoxide_html_results/diazoxide_syringe/index.html?calibrationId=calt0om200.html]

Stability Study Cruncher


### Calibration Id: calt0om200

Slope: 374038 1/mg/mL (r2 = 0.99997, n = 15).

|  |  |  |  |  |  |  |  |  |  |  |  |  |  |  |  |  |  |  |  |  |  |  |  |  |  |  |  |  |  |  |  |  |  |  |  |  |  |  |  |  |  |  |  |  |  |  |  |
| --- | --- | --- | --- | --- | --- | --- | --- | --- | --- | --- | --- | --- | --- | --- | --- | --- | --- | --- | --- | --- | --- | --- | --- | --- | --- | --- | --- | --- | --- | --- | --- | --- | --- | --- | --- | --- | --- | --- | --- | --- | --- | --- | --- | --- | --- | --- | --- |
| Input String | Conc | Area |||  |  |  |  |  |  |  |  |  |  |  |  |  |  |  |  |  |  |  |  |  |  |  |  |  |  |  |  |  |  |  |  |  |  |  |  |  |  |  |  |  |  |  |  |  |
| --- | --- | --- | --- | --- | --- | --- | --- | --- | --- | --- | --- | --- | --- | --- | --- | --- | --- | --- | --- | --- | --- | --- | --- | --- | --- | --- | --- | --- | --- | --- | --- | --- | --- | --- | --- | --- | --- | --- | --- | --- | --- | --- | --- | --- |
| diazoxide\_STD000;0;0;calt0om200;calibration | 0.00 | 0 || diazoxide\_STD025;1896987;5;calt0om200;calibration | 5.00 | 1896987 || diazoxide\_STD050;3760298;10;calt0om200;calibration | 10.00 | 3760298 || diazoxide\_STD075;5639811;15;calt0om200;calibration | 15.00 | 5639811 || diazoxide\_STD100;7454879;20;calt0om200;calibration | 20.00 | 7454879 || diazoxide\_STD000;0;0;calt0om200;calibration | 0.00 | 0 || diazoxide\_STD025;1895076;5;calt0om200;calibration | 5.00 | 1895076 || diazoxide\_STD050;3769921;10;calt0om200;calibration | 10.00 | 3769921 || diazoxide\_STD075;5630254;15;calt0om200;calibration | 15.00 | 5630254 || diazoxide\_STD100;7443879;20;calt0om200;calibration | 20.00 | 7443879 || diazoxide\_STD000;0;0;calt0om200;calibration | 0.00 | 0 || diazoxide\_STD025;1895141;5;calt0om200;calibration | 5.00 | 1895141 || diazoxide\_STD050;3761030;10;calt0om200;calibration | 10.00 | 3761030 || diazoxide\_STD075;5627350;15;calt0om200;calibration | 15.00 | 5627350 || diazoxide\_STD100;7440100;20;calt0om200;calibration | 20.00 | 7440100 |
