# Supplementary material for: Stability of Diazoxide in Extemporaneously Compounded Oral Suspensions
Source: PLoS One. 2016 Oct 11;11(10):e0164577. doi: 10.1371/journal.pone.0164577 (PMC5058506; doi:10.1371/journal.pone.0164577)
Supplement: S2 Appendix — Archive containing the HPLC stability results as browsable html pages. (ZIP) [file pone.0164577.s002.zip › diazoxide_html_results/diazoxide_syringe/index.html?preparation=tablet-oralmixsf&lot=a&condition=syringe-5&time=90.html]

Stability Study Cruncher


### Preparation: tablet-oralmixsf, Lot: a, Condition: syringe-5, Time: 90

Assay (mg/mL): 10.23 ± 0.57 (n = 3);
Assay (%TZ): 101.9 ± 5.7 (n = 3).

| Input String | Area | Cal Id | Cal Slope | Assay | Assay TZ | Assay %TZ |  |
| --- | --- | --- | --- | --- | --- | --- | --- |
| diazoxide\_tablet-oralmixsf\_a\_syringe-5\_90;3502252;;cal60sf210;stability | 3502252 | cal60sf210 | 358176 | 9.78 | 10.05 | 97.3 | calibration, time zero |
| diazoxide\_tablet-oralmixsf\_a\_syringe-5\_90;3598494;;cal60sf210;stability | 3598494 | cal60sf210 | 358176 | 10.05 | 10.05 | 100.0 | calibration, time zero |
| diazoxide\_tablet-oralmixsf\_a\_syringe-5\_90;3896509;;cal60sf210;stability | 3896509 | cal60sf210 | 358176 | 10.88 | 10.05 | 108.3 | calibration, time zero |
