# Supplementary material for: Stability of Diazoxide in Extemporaneously Compounded Oral Suspensions
Source: PLoS One. 2016 Oct 11;11(10):e0164577. doi: 10.1371/journal.pone.0164577 (PMC5058506; doi:10.1371/journal.pone.0164577)
Supplement: S2 Appendix — Archive containing the HPLC stability results as browsable html pages. (ZIP) [file pone.0164577.s002.zip › diazoxide_html_results/diazoxide_syringe/index.html?preparation=bulk-oralmixsf&lot=a&condition=syringe-25&time=7.html]

Stability Study Cruncher


### Preparation: bulk-oralmixsf, Lot: a, Condition: syringe-25, Time: 7

Assay (mg/mL): 9.18 ± 0.14 (n = 3);
Assay (%TZ): 92.0 ± 1.4 (n = 3).

| Input String | Area | Cal Id | Cal Slope | Assay | Assay TZ | Assay %TZ |  |
| --- | --- | --- | --- | --- | --- | --- | --- |
| diazoxide\_bulk-oralmixsf\_a\_syringe-25\_7;3314846;;cal7sf210;stability | 3314846 | cal7sf210 | 355486 | 9.32 | 9.98 | 93.5 | calibration, time zero |
| diazoxide\_bulk-oralmixsf\_a\_syringe-25\_7;3376301;;cal7sf200;stability | 3376301 | cal7sf200 | 373260 | 9.05 | 9.98 | 90.7 | calibration, time zero |
| diazoxide\_bulk-oralmixsf\_a\_syringe-25\_7;3418555;;cal7sf200;stability | 3418555 | cal7sf200 | 373260 | 9.16 | 9.98 | 91.8 | calibration, time zero |
