# Supplementary material for: Stability of Diazoxide in Extemporaneously Compounded Oral Suspensions
Source: PLoS One. 2016 Oct 11;11(10):e0164577. doi: 10.1371/journal.pone.0164577 (PMC5058506; doi:10.1371/journal.pone.0164577)
Supplement: S2 Appendix — Archive containing the HPLC stability results as browsable html pages. (ZIP) [file pone.0164577.s002.zip › diazoxide_html_results/diazoxide_syringe/index.html?preparation=bulk-oralmixsf&lot=a&condition=syringe-25&time=30.html]

Stability Study Cruncher


### Preparation: bulk-oralmixsf, Lot: a, Condition: syringe-25, Time: 30

Assay (mg/mL): 9.43 ± 0.23 (n = 3);
Assay (%TZ): 94.5 ± 2.3 (n = 3).

| Input String | Area | Cal Id | Cal Slope | Assay | Assay TZ | Assay %TZ |  |
| --- | --- | --- | --- | --- | --- | --- | --- |
| diazoxide\_bulk-oralmixsf\_a\_syringe-25\_30;3407787;;cal30sf210;stability | 3407787 | cal30sf210 | 358295 | 9.51 | 9.98 | 95.3 | calibration, time zero |
| diazoxide\_bulk-oralmixsf\_a\_syringe-25\_30;3282614;;cal30sf210;stability | 3282614 | cal30sf210 | 358295 | 9.16 | 9.98 | 91.8 | calibration, time zero |
| diazoxide\_bulk-oralmixsf\_a\_syringe-25\_30;3441253;;cal30sf210;stability | 3441253 | cal30sf210 | 358295 | 9.60 | 9.98 | 96.3 | calibration, time zero |
