# Supplementary material for: Stability of Diazoxide in Extemporaneously Compounded Oral Suspensions
Source: PLoS One. 2016 Oct 11;11(10):e0164577. doi: 10.1371/journal.pone.0164577 (PMC5058506; doi:10.1371/journal.pone.0164577)
Supplement: S2 Appendix — Archive containing the HPLC stability results as browsable html pages. (ZIP) [file pone.0164577.s002.zip › diazoxide_html_results/diazoxide_syringe/index.html?calibrationId=cal7om200.html]

Stability Study Cruncher


### Calibration Id: cal7om200

Slope: 373935 1/mg/mL (r2 = 0.99997, n = 15).

|  |  |  |  |  |  |  |  |  |  |  |  |  |  |  |  |  |  |  |  |  |  |  |  |  |  |  |  |  |  |  |  |  |  |  |  |  |  |  |  |  |  |  |  |  |  |  |  |
| --- | --- | --- | --- | --- | --- | --- | --- | --- | --- | --- | --- | --- | --- | --- | --- | --- | --- | --- | --- | --- | --- | --- | --- | --- | --- | --- | --- | --- | --- | --- | --- | --- | --- | --- | --- | --- | --- | --- | --- | --- | --- | --- | --- | --- | --- | --- | --- |
| Input String | Conc | Area |||  |  |  |  |  |  |  |  |  |  |  |  |  |  |  |  |  |  |  |  |  |  |  |  |  |  |  |  |  |  |  |  |  |  |  |  |  |  |  |  |  |  |  |  |  |
| --- | --- | --- | --- | --- | --- | --- | --- | --- | --- | --- | --- | --- | --- | --- | --- | --- | --- | --- | --- | --- | --- | --- | --- | --- | --- | --- | --- | --- | --- | --- | --- | --- | --- | --- | --- | --- | --- | --- | --- | --- | --- | --- | --- | --- |
| diazoxide\_STD000;0;0;cal7om200;calibration | 0.00 | 0 || diazoxide\_STD025;1897442;5;cal7om200;calibration | 5.00 | 1897442 || diazoxide\_STD050;3761639;10;cal7om200;calibration | 10.00 | 3761639 || diazoxide\_STD075;5624782;15;cal7om200;calibration | 15.00 | 5624782 || diazoxide\_STD100;7441685;20;cal7om200;calibration | 20.00 | 7441685 || diazoxide\_STD000;0;0;cal7om200;calibration | 0.00 | 0 || diazoxide\_STD025;1897609;5;cal7om200;calibration | 5.00 | 1897609 || diazoxide\_STD050;3764206;10;cal7om200;calibration | 10.00 | 3764206 || diazoxide\_STD075;5627826;15;cal7om200;calibration | 15.00 | 5627826 || diazoxide\_STD100;7449002;20;cal7om200;calibration | 20.00 | 7449002 || diazoxide\_STD000;0;0;cal7om200;calibration | 0.00 | 0 || diazoxide\_STD025;1898280;5;cal7om200;calibration | 5.00 | 1898280 || diazoxide\_STD050;3762487;10;cal7om200;calibration | 10.00 | 3762487 || diazoxide\_STD075;5630060;15;cal7om200;calibration | 15.00 | 5630060 || diazoxide\_STD100;7447547;20;cal7om200;calibration | 20.00 | 7447547 |
