# Supplementary material for: Stability of Diazoxide in Extemporaneously Compounded Oral Suspensions
Source: PLoS One. 2016 Oct 11;11(10):e0164577. doi: 10.1371/journal.pone.0164577 (PMC5058506; doi:10.1371/journal.pone.0164577)
Supplement: S2 Appendix — Archive containing the HPLC stability results as browsable html pages. (ZIP) [file pone.0164577.s002.zip › diazoxide_html_results/diazoxide_syringe/index.html?preparation=bulk-oralmixsf&lot=a&condition=syringe-25&time=90.html]

Stability Study Cruncher


### Preparation: bulk-oralmixsf, Lot: a, Condition: syringe-25, Time: 90

Assay (mg/mL): 9.53 ± 0.26 (n = 3);
Assay (%TZ): 95.6 ± 2.6 (n = 3).

| Input String | Area | Cal Id | Cal Slope | Assay | Assay TZ | Assay %TZ |  |
| --- | --- | --- | --- | --- | --- | --- | --- |
| diazoxide\_bulk-oralmixsf\_a\_syringe-25\_90;3520747;;cal60sf210;stability | 3520747 | cal60sf210 | 358176 | 9.83 | 9.98 | 98.5 | calibration, time zero |
| diazoxide\_bulk-oralmixsf\_a\_syringe-25\_90;3369453;;cal60sf210;stability | 3369453 | cal60sf210 | 358176 | 9.41 | 9.98 | 94.3 | calibration, time zero |
| diazoxide\_bulk-oralmixsf\_a\_syringe-25\_90;3354427;;cal60sf210;stability | 3354427 | cal60sf210 | 358176 | 9.37 | 9.98 | 93.9 | calibration, time zero |
