# Supplementary material for: Stability of Diazoxide in Extemporaneously Compounded Oral Suspensions
Source: PLoS One. 2016 Oct 11;11(10):e0164577. doi: 10.1371/journal.pone.0164577 (PMC5058506; doi:10.1371/journal.pone.0164577)
Supplement: S2 Appendix — Archive containing the HPLC stability results as browsable html pages. (ZIP) [file pone.0164577.s002.zip › diazoxide_html_results/diazoxide_syringe/index.html?preparation=tablet-oralmixsf&lot=a&condition=syringe-25&time=7.html]

Stability Study Cruncher


### Preparation: tablet-oralmixsf, Lot: a, Condition: syringe-25, Time: 7

Assay (mg/mL): 9.71 ± 0.20 (n = 3);
Assay (%TZ): 96.6 ± 2.0 (n = 3).

| Input String | Area | Cal Id | Cal Slope | Assay | Assay TZ | Assay %TZ |  |
| --- | --- | --- | --- | --- | --- | --- | --- |
| diazoxide\_tablet-oralmixsf\_a\_syringe-25\_7;3710210;;cal7sf200;stability | 3710210 | cal7sf200 | 373260 | 9.94 | 10.05 | 98.9 | calibration, time zero |
| diazoxide\_tablet-oralmixsf\_a\_syringe-25\_7;3572597;;cal7sf200;stability | 3572597 | cal7sf200 | 373260 | 9.57 | 10.05 | 95.3 | calibration, time zero |
| diazoxide\_tablet-oralmixsf\_a\_syringe-25\_7;3588434;;cal7sf200;stability | 3588434 | cal7sf200 | 373260 | 9.61 | 10.05 | 95.7 | calibration, time zero |
