# Supplementary material for: Stability of Diazoxide in Extemporaneously Compounded Oral Suspensions
Source: PLoS One. 2016 Oct 11;11(10):e0164577. doi: 10.1371/journal.pone.0164577 (PMC5058506; doi:10.1371/journal.pone.0164577)
Supplement: S2 Appendix — Archive containing the HPLC stability results as browsable html pages. (ZIP) [file pone.0164577.s002.zip › diazoxide_html_results/diazoxide_syringe/index.html?preparation=tablet-oralmixsf&lot=a&condition=syringe-25&time=14.html]

Stability Study Cruncher


### Preparation: tablet-oralmixsf, Lot: a, Condition: syringe-25, Time: 14

Assay (mg/mL): 9.21 ± 0.12 (n = 3);
Assay (%TZ): 91.7 ± 1.2 (n = 3).

| Input String | Area | Cal Id | Cal Slope | Assay | Assay TZ | Assay %TZ |  |
| --- | --- | --- | --- | --- | --- | --- | --- |
| diazoxide\_tablet-oralmixsf\_a\_syringe-25\_14;3342311;;cal14sf210;stability | 3342311 | cal14sf210 | 359483 | 9.30 | 10.05 | 92.5 | calibration, time zero |
| diazoxide\_tablet-oralmixsf\_a\_syringe-25\_14;3327487;;cal14sf210;stability | 3327487 | cal14sf210 | 359483 | 9.26 | 10.05 | 92.1 | calibration, time zero |
| diazoxide\_tablet-oralmixsf\_a\_syringe-25\_14;3263122;;cal14sf210;stability | 3263122 | cal14sf210 | 359483 | 9.08 | 10.05 | 90.3 | calibration, time zero |
