# Supplementary material for: Stability of Diazoxide in Extemporaneously Compounded Oral Suspensions
Source: PLoS One. 2016 Oct 11;11(10):e0164577. doi: 10.1371/journal.pone.0164577 (PMC5058506; doi:10.1371/journal.pone.0164577)
Supplement: S2 Appendix — Archive containing the HPLC stability results as browsable html pages. (ZIP) [file pone.0164577.s002.zip › diazoxide_html_results/diazoxide_syringe/index.html?calibrationId=cal14om210.html]

Stability Study Cruncher


### Calibration Id: cal14om210

Slope: 358223 1/mg/mL (r2 = 0.99998, n = 15).

|  |  |  |  |  |  |  |  |  |  |  |  |  |  |  |  |  |  |  |  |  |  |  |  |  |  |  |  |  |  |  |  |  |  |  |  |  |  |  |  |  |  |  |  |  |  |  |  |
| --- | --- | --- | --- | --- | --- | --- | --- | --- | --- | --- | --- | --- | --- | --- | --- | --- | --- | --- | --- | --- | --- | --- | --- | --- | --- | --- | --- | --- | --- | --- | --- | --- | --- | --- | --- | --- | --- | --- | --- | --- | --- | --- | --- | --- | --- | --- | --- |
| Input String | Conc | Area |||  |  |  |  |  |  |  |  |  |  |  |  |  |  |  |  |  |  |  |  |  |  |  |  |  |  |  |  |  |  |  |  |  |  |  |  |  |  |  |  |  |  |  |  |  |
| --- | --- | --- | --- | --- | --- | --- | --- | --- | --- | --- | --- | --- | --- | --- | --- | --- | --- | --- | --- | --- | --- | --- | --- | --- | --- | --- | --- | --- | --- | --- | --- | --- | --- | --- | --- | --- | --- | --- | --- | --- | --- | --- | --- | --- |
| diazoxide\_STD000;0;0;cal14om210;calibration | 0.00 | 0 || diazoxide\_STD025;1908500;5.25;cal14om210;calibration | 5.25 | 1908500 || diazoxide\_STD050;3779826;10.5;cal14om210;calibration | 10.50 | 3779826 || diazoxide\_STD075;5655966;15.75;cal14om210;calibration | 15.75 | 5655966 || diazoxide\_STD100;7489837;21;cal14om210;calibration | 21.00 | 7489837 || diazoxide\_STD000;0;0;cal14om210;calibration | 0.00 | 0 || diazoxide\_STD025;1906737;5.25;cal14om210;calibration | 5.25 | 1906737 || diazoxide\_STD050;3780821;10.5;cal14om210;calibration | 10.50 | 3780821 || diazoxide\_STD075;5659152;15.75;cal14om210;calibration | 15.75 | 5659152 || diazoxide\_STD100;7496711;21;cal14om210;calibration | 21.00 | 7496711 || diazoxide\_STD000;0;0;cal14om210;calibration | 0.00 | 0 || diazoxide\_STD025;1907425;5.25;cal14om210;calibration | 5.25 | 1907425 || diazoxide\_STD050;3779919;10.5;cal14om210;calibration | 10.50 | 3779919 || diazoxide\_STD075;5658036;15.75;cal14om210;calibration | 15.75 | 5658036 || diazoxide\_STD100;7497771;21;cal14om210;calibration | 21.00 | 7497771 |
