# Supplementary material for: Stability of Diazoxide in Extemporaneously Compounded Oral Suspensions
Source: PLoS One. 2016 Oct 11;11(10):e0164577. doi: 10.1371/journal.pone.0164577 (PMC5058506; doi:10.1371/journal.pone.0164577)
Supplement: S2 Appendix — Archive containing the HPLC stability results as browsable html pages. (ZIP) [file pone.0164577.s002.zip › diazoxide_html_results/diazoxide_syringe/index.html?preparation=tablet-oralmixsf&lot=a&condition=syringe-25&time=45.html]

Stability Study Cruncher


### Preparation: tablet-oralmixsf, Lot: a, Condition: syringe-25, Time: 45

Assay (mg/mL): 9.82 ± 0.37 (n = 3);
Assay (%TZ): 97.8 ± 3.7 (n = 3).

| Input String | Area | Cal Id | Cal Slope | Assay | Assay TZ | Assay %TZ |  |
| --- | --- | --- | --- | --- | --- | --- | --- |
| diazoxide\_tablet-oralmixsf\_a\_syringe-25\_45;3409578;;cal30sf210;stability | 3409578 | cal30sf210 | 358295 | 9.52 | 10.05 | 94.7 | calibration, time zero |
| diazoxide\_tablet-oralmixsf\_a\_syringe-25\_45;3483394;;cal30sf210;stability | 3483394 | cal30sf210 | 358295 | 9.72 | 10.05 | 96.8 | calibration, time zero |
| diazoxide\_tablet-oralmixsf\_a\_syringe-25\_45;3665179;;cal30sf210;stability | 3665179 | cal30sf210 | 358295 | 10.23 | 10.05 | 101.8 | calibration, time zero |
