# Supplementary material for: Stability of Diazoxide in Extemporaneously Compounded Oral Suspensions
Source: PLoS One. 2016 Oct 11;11(10):e0164577. doi: 10.1371/journal.pone.0164577 (PMC5058506; doi:10.1371/journal.pone.0164577)
Supplement: S2 Appendix — Archive containing the HPLC stability results as browsable html pages. (ZIP) [file pone.0164577.s002.zip › diazoxide_html_results/diazoxide_syringe/index.html?preparation=tablet-oralmixsf&lot=a&condition=syringe-25&time=90.html]

Stability Study Cruncher


### Preparation: tablet-oralmixsf, Lot: a, Condition: syringe-25, Time: 90

Assay (mg/mL): 10.28 ± 0.32 (n = 3);
Assay (%TZ): 102.3 ± 3.2 (n = 3).

| Input String | Area | Cal Id | Cal Slope | Assay | Assay TZ | Assay %TZ |  |
| --- | --- | --- | --- | --- | --- | --- | --- |
| diazoxide\_tablet-oralmixsf\_a\_syringe-25\_90;3804712;;cal60sf210;stability | 3804712 | cal60sf210 | 358176 | 10.62 | 10.05 | 105.7 | calibration, time zero |
| diazoxide\_tablet-oralmixsf\_a\_syringe-25\_90;3573794;;cal60sf210;stability | 3573794 | cal60sf210 | 358176 | 9.98 | 10.05 | 99.3 | calibration, time zero |
| diazoxide\_tablet-oralmixsf\_a\_syringe-25\_90;3669519;;cal60sf210;stability | 3669519 | cal60sf210 | 358176 | 10.25 | 10.05 | 102.0 | calibration, time zero |
