# Supplementary material for: Stability of Diazoxide in Extemporaneously Compounded Oral Suspensions
Source: PLoS One. 2016 Oct 11;11(10):e0164577. doi: 10.1371/journal.pone.0164577 (PMC5058506; doi:10.1371/journal.pone.0164577)
Supplement: S2 Appendix — Archive containing the HPLC stability results as browsable html pages. (ZIP) [file pone.0164577.s002.zip › diazoxide_html_results/diazoxide_syringe/index.html?calibrationId=cal75om210.html]

Stability Study Cruncher


### Calibration Id: cal75om210

Slope: 358017 1/mg/mL (r2 = 0.99998, n = 15).

|  |  |  |  |  |  |  |  |  |  |  |  |  |  |  |  |  |  |  |  |  |  |  |  |  |  |  |  |  |  |  |  |  |  |  |  |  |  |  |  |  |  |  |  |  |  |  |  |
| --- | --- | --- | --- | --- | --- | --- | --- | --- | --- | --- | --- | --- | --- | --- | --- | --- | --- | --- | --- | --- | --- | --- | --- | --- | --- | --- | --- | --- | --- | --- | --- | --- | --- | --- | --- | --- | --- | --- | --- | --- | --- | --- | --- | --- | --- | --- | --- |
| Input String | Conc | Area |||  |  |  |  |  |  |  |  |  |  |  |  |  |  |  |  |  |  |  |  |  |  |  |  |  |  |  |  |  |  |  |  |  |  |  |  |  |  |  |  |  |  |  |  |  |
| --- | --- | --- | --- | --- | --- | --- | --- | --- | --- | --- | --- | --- | --- | --- | --- | --- | --- | --- | --- | --- | --- | --- | --- | --- | --- | --- | --- | --- | --- | --- | --- | --- | --- | --- | --- | --- | --- | --- | --- | --- | --- | --- | --- | --- |
| diazoxide\_STD000;0;0;cal75om210;calibration | 0.00 | 0 || diazoxide\_STD025;1905377;5.25;cal75om210;calibration | 5.25 | 1905377 || diazoxide\_STD050;3778944;10.5;cal75om210;calibration | 10.50 | 3778944 || diazoxide\_STD075;5651272;15.75;cal75om210;calibration | 15.75 | 5651272 || diazoxide\_STD100;7483575;21;cal75om210;calibration | 21.00 | 7483575 || diazoxide\_STD000;0;0;cal75om210;calibration | 0.00 | 0 || diazoxide\_STD025;1906591;5.25;cal75om210;calibration | 5.25 | 1906591 || diazoxide\_STD050;3779892;10.5;cal75om210;calibration | 10.50 | 3779892 || diazoxide\_STD075;5653836;15.75;cal75om210;calibration | 15.75 | 5653836 || diazoxide\_STD100;7492414;21;cal75om210;calibration | 21.00 | 7492414 || diazoxide\_STD000;0;0;cal75om210;calibration | 0.00 | 0 || diazoxide\_STD025;1907167;5.25;cal75om210;calibration | 5.25 | 1907167 || diazoxide\_STD050;3781070;10.5;cal75om210;calibration | 10.50 | 3781070 || diazoxide\_STD075;5655346;15.75;cal75om210;calibration | 15.75 | 5655346 || diazoxide\_STD100;7494719;21;cal75om210;calibration | 21.00 | 7494719 |
