# Supplementary material for: Stability of Diazoxide in Extemporaneously Compounded Oral Suspensions
Source: PLoS One. 2016 Oct 11;11(10):e0164577. doi: 10.1371/journal.pone.0164577 (PMC5058506; doi:10.1371/journal.pone.0164577)
Supplement: S2 Appendix — Archive containing the HPLC stability results as browsable html pages. (ZIP) [file pone.0164577.s002.zip › diazoxide_html_results/diazoxide_syringe/index.html?calibrationId=cal7sf200.html]

Stability Study Cruncher


### Calibration Id: cal7sf200

Slope: 373260 1/mg/mL (r2 = 0.99996, n = 15).

|  |  |  |  |  |  |  |  |  |  |  |  |  |  |  |  |  |  |  |  |  |  |  |  |  |  |  |  |  |  |  |  |  |  |  |  |  |  |  |  |  |  |  |  |  |  |  |  |
| --- | --- | --- | --- | --- | --- | --- | --- | --- | --- | --- | --- | --- | --- | --- | --- | --- | --- | --- | --- | --- | --- | --- | --- | --- | --- | --- | --- | --- | --- | --- | --- | --- | --- | --- | --- | --- | --- | --- | --- | --- | --- | --- | --- | --- | --- | --- | --- |
| Input String | Conc | Area |||  |  |  |  |  |  |  |  |  |  |  |  |  |  |  |  |  |  |  |  |  |  |  |  |  |  |  |  |  |  |  |  |  |  |  |  |  |  |  |  |  |  |  |  |  |
| --- | --- | --- | --- | --- | --- | --- | --- | --- | --- | --- | --- | --- | --- | --- | --- | --- | --- | --- | --- | --- | --- | --- | --- | --- | --- | --- | --- | --- | --- | --- | --- | --- | --- | --- | --- | --- | --- | --- | --- | --- | --- | --- | --- | --- |
| diazoxide\_STD000;0;0;cal7sf200;calibration | 0.00 | 0 || diazoxide\_STD025;1877800;5;cal7sf200;calibration | 5.00 | 1877800 || diazoxide\_STD050;3743826;10;cal7sf200;calibration | 10.00 | 3743826 || diazoxide\_STD075;5643822;15;cal7sf200;calibration | 15.00 | 5643822 || diazoxide\_STD100;7418398;20;cal7sf200;calibration | 20.00 | 7418398 || diazoxide\_STD000;0;0;cal7sf200;calibration | 0.00 | 0 || diazoxide\_STD025;1875259;5;cal7sf200;calibration | 5.00 | 1875259 || diazoxide\_STD050;3742278;10;cal7sf200;calibration | 10.00 | 3742278 || diazoxide\_STD075;5642303;15;cal7sf200;calibration | 15.00 | 5642303 || diazoxide\_STD100;7422579;20;cal7sf200;calibration | 20.00 | 7422579 || diazoxide\_STD000;0;0;cal7sf200;calibration | 0.00 | 0 || diazoxide\_STD025;1875021;5;cal7sf200;calibration | 5.00 | 1875021 || diazoxide\_STD050;3743907;10;cal7sf200;calibration | 10.00 | 3743907 || diazoxide\_STD075;5646417;15;cal7sf200;calibration | 15.00 | 5646417 || diazoxide\_STD100;7429429;20;cal7sf200;calibration | 20.00 | 7429429 |
