# Supplementary material for: Stability of Diazoxide in Extemporaneously Compounded Oral Suspensions
Source: PLoS One. 2016 Oct 11;11(10):e0164577. doi: 10.1371/journal.pone.0164577 (PMC5058506; doi:10.1371/journal.pone.0164577)
Supplement: S2 Appendix — Archive containing the HPLC stability results as browsable html pages. (ZIP) [file pone.0164577.s002.zip › diazoxide_html_results/diazoxide_syringe/index.html?calibrationId=cal14sf210.html]

Stability Study Cruncher


### Calibration Id: cal14sf210

Slope: 359483 1/mg/mL (r2 = 1.00000, n = 15).

|  |  |  |  |  |  |  |  |  |  |  |  |  |  |  |  |  |  |  |  |  |  |  |  |  |  |  |  |  |  |  |  |  |  |  |  |  |  |  |  |  |  |  |  |  |  |  |  |
| --- | --- | --- | --- | --- | --- | --- | --- | --- | --- | --- | --- | --- | --- | --- | --- | --- | --- | --- | --- | --- | --- | --- | --- | --- | --- | --- | --- | --- | --- | --- | --- | --- | --- | --- | --- | --- | --- | --- | --- | --- | --- | --- | --- | --- | --- | --- | --- |
| Input String | Conc | Area |||  |  |  |  |  |  |  |  |  |  |  |  |  |  |  |  |  |  |  |  |  |  |  |  |  |  |  |  |  |  |  |  |  |  |  |  |  |  |  |  |  |  |  |  |  |
| --- | --- | --- | --- | --- | --- | --- | --- | --- | --- | --- | --- | --- | --- | --- | --- | --- | --- | --- | --- | --- | --- | --- | --- | --- | --- | --- | --- | --- | --- | --- | --- | --- | --- | --- | --- | --- | --- | --- | --- | --- | --- | --- | --- | --- |
| diazoxide\_STD000\_SF;0;0;cal14sf210;calibration | 0.00 | 0 || diazoxide\_STD025\_SF;1885787;5.25;cal14sf210;calibration | 5.25 | 1885787 || diazoxide\_STD050\_SF;3769325;10.5;cal14sf210;calibration | 10.50 | 3769325 || diazoxide\_STD075\_SF;5653475;15.75;cal14sf210;calibration | 15.75 | 5653475 || diazoxide\_STD100\_SF;7554444;21;cal14sf210;calibration | 21.00 | 7554444 || diazoxide\_STD000\_SF;0;0;cal14sf210;calibration | 0.00 | 0 || diazoxide\_STD025\_SF;1887157;5.25;cal14sf210;calibration | 5.25 | 1887157 || diazoxide\_STD050\_SF;3763717;10.5;cal14sf210;calibration | 10.50 | 3763717 || diazoxide\_STD075\_SF;5660069;15.75;cal14sf210;calibration | 15.75 | 5660069 || diazoxide\_STD100\_SF;7557256;21;cal14sf210;calibration | 21.00 | 7557256 || diazoxide\_STD000\_SF;0;0;cal14sf210;calibration | 0.00 | 0 || diazoxide\_STD025\_SF;1886979;5.25;cal14sf210;calibration | 5.25 | 1886979 || diazoxide\_STD050\_SF;3765360;10.5;cal14sf210;calibration | 10.50 | 3765360 || diazoxide\_STD075\_SF;5655153;15.75;cal14sf210;calibration | 15.75 | 5655153 || diazoxide\_STD100\_SF;7561614;21;cal14sf210;calibration | 21.00 | 7561614 |
