# Supplementary material for: Stability of Diazoxide in Extemporaneously Compounded Oral Suspensions
Source: PLoS One. 2016 Oct 11;11(10):e0164577. doi: 10.1371/journal.pone.0164577 (PMC5058506; doi:10.1371/journal.pone.0164577)
Supplement: S2 Appendix — Archive containing the HPLC stability results as browsable html pages. (ZIP) [file pone.0164577.s002.zip › diazoxide_html_results/diazoxide_syringe/index.html?calibrationId=cal30sf210.html]

Stability Study Cruncher


### Calibration Id: cal30sf210

Slope: 358295 1/mg/mL (r2 = 1.00000, n = 15).

|  |  |  |  |  |  |  |  |  |  |  |  |  |  |  |  |  |  |  |  |  |  |  |  |  |  |  |  |  |  |  |  |  |  |  |  |  |  |  |  |  |  |  |  |  |  |  |  |
| --- | --- | --- | --- | --- | --- | --- | --- | --- | --- | --- | --- | --- | --- | --- | --- | --- | --- | --- | --- | --- | --- | --- | --- | --- | --- | --- | --- | --- | --- | --- | --- | --- | --- | --- | --- | --- | --- | --- | --- | --- | --- | --- | --- | --- | --- | --- | --- |
| Input String | Conc | Area |||  |  |  |  |  |  |  |  |  |  |  |  |  |  |  |  |  |  |  |  |  |  |  |  |  |  |  |  |  |  |  |  |  |  |  |  |  |  |  |  |  |  |  |  |  |
| --- | --- | --- | --- | --- | --- | --- | --- | --- | --- | --- | --- | --- | --- | --- | --- | --- | --- | --- | --- | --- | --- | --- | --- | --- | --- | --- | --- | --- | --- | --- | --- | --- | --- | --- | --- | --- | --- | --- | --- | --- | --- | --- | --- | --- |
| diazoxide\_STD000\_SF;0;0;cal30sf210;calibration | 0.00 | 0 || diazoxide\_STD025\_SF;1880843;5.25;cal30sf210;calibration | 5.25 | 1880843 || diazoxide\_STD050\_SF;3752941;10.5;cal30sf210;calibration | 10.50 | 3752941 || diazoxide\_STD075\_SF;5629240;15.75;cal30sf210;calibration | 15.75 | 5629240 || diazoxide\_STD100\_SF;7530878;21;cal30sf210;calibration | 21.00 | 7530878 || diazoxide\_STD000\_SF;0;0;cal30sf210;calibration | 0.00 | 0 || diazoxide\_STD025\_SF;1879201;5.25;cal30sf210;calibration | 5.25 | 1879201 || diazoxide\_STD050\_SF;3752907;10.5;cal30sf210;calibration | 10.50 | 3752907 || diazoxide\_STD075\_SF;5634757;15.75;cal30sf210;calibration | 15.75 | 5634757 || diazoxide\_STD100\_SF;7537758;21;cal30sf210;calibration | 21.00 | 7537758 || diazoxide\_STD000\_SF;0;0;cal30sf210;calibration | 0.00 | 0 || diazoxide\_STD025\_SF;1880168;5.25;cal30sf210;calibration | 5.25 | 1880168 || diazoxide\_STD050\_SF;3753166;10.5;cal30sf210;calibration | 10.50 | 3753166 || diazoxide\_STD075\_SF;5634198;15.75;cal30sf210;calibration | 15.75 | 5634198 || diazoxide\_STD100\_SF;7541762;21;cal30sf210;calibration | 21.00 | 7541762 |
