# Supplementary material for: Stability of Diazoxide in Extemporaneously Compounded Oral Suspensions
Source: PLoS One. 2016 Oct 11;11(10):e0164577. doi: 10.1371/journal.pone.0164577 (PMC5058506; doi:10.1371/journal.pone.0164577)
Supplement: S2 Appendix — Archive containing the HPLC stability results as browsable html pages. (ZIP) [file pone.0164577.s002.zip › diazoxide_html_results/diazoxide_syringe/index.html?calibrationId=cal60sf210.html]

Stability Study Cruncher


### Calibration Id: cal60sf210

Slope: 358176 1/mg/mL (r2 = 1.00000, n = 15).

|  |  |  |  |  |  |  |  |  |  |  |  |  |  |  |  |  |  |  |  |  |  |  |  |  |  |  |  |  |  |  |  |  |  |  |  |  |  |  |  |  |  |  |  |  |  |  |  |
| --- | --- | --- | --- | --- | --- | --- | --- | --- | --- | --- | --- | --- | --- | --- | --- | --- | --- | --- | --- | --- | --- | --- | --- | --- | --- | --- | --- | --- | --- | --- | --- | --- | --- | --- | --- | --- | --- | --- | --- | --- | --- | --- | --- | --- | --- | --- | --- |
| Input String | Conc | Area |||  |  |  |  |  |  |  |  |  |  |  |  |  |  |  |  |  |  |  |  |  |  |  |  |  |  |  |  |  |  |  |  |  |  |  |  |  |  |  |  |  |  |  |  |  |
| --- | --- | --- | --- | --- | --- | --- | --- | --- | --- | --- | --- | --- | --- | --- | --- | --- | --- | --- | --- | --- | --- | --- | --- | --- | --- | --- | --- | --- | --- | --- | --- | --- | --- | --- | --- | --- | --- | --- | --- | --- | --- | --- | --- | --- |
| diazoxide\_STD000\_SF;0;0;cal60sf210;calibration | 0.00 | 0 || diazoxide\_STD025\_SF;1882564;5.25;cal60sf210;calibration | 5.25 | 1882564 || diazoxide\_STD050\_SF;3755970;10.5;cal60sf210;calibration | 10.50 | 3755970 || diazoxide\_STD075\_SF;5628766;15.75;cal60sf210;calibration | 15.75 | 5628766 || diazoxide\_STD100\_SF;7519361;21;cal60sf210;calibration | 21.00 | 7519361 || diazoxide\_STD000\_SF;0;0;cal60sf210;calibration | 0.00 | 0 || diazoxide\_STD025\_SF;1881801;5.25;cal60sf210;calibration | 5.25 | 1881801 || diazoxide\_STD050\_SF;3757104;10.5;cal60sf210;calibration | 10.50 | 3757104 || diazoxide\_STD075\_SF;5632636;15.75;cal60sf210;calibration | 15.75 | 5632636 || diazoxide\_STD100\_SF;7531705;21;cal60sf210;calibration | 21.00 | 7531705 || diazoxide\_STD000\_SF;0;0;cal60sf210;calibration | 0.00 | 0 || diazoxide\_STD025\_SF;1883466;5.25;cal60sf210;calibration | 5.25 | 1883466 || diazoxide\_STD050\_SF;3758425;10.5;cal60sf210;calibration | 10.50 | 3758425 || diazoxide\_STD075\_SF;5635899;15.75;cal60sf210;calibration | 15.75 | 5635899 || diazoxide\_STD100\_SF;7537840;21;cal60sf210;calibration | 21.00 | 7537840 |
