# Supplementary material for: Stability of Diazoxide in Extemporaneously Compounded Oral Suspensions
Source: PLoS One. 2016 Oct 11;11(10):e0164577. doi: 10.1371/journal.pone.0164577 (PMC5058506; doi:10.1371/journal.pone.0164577)
Supplement: S2 Appendix — Archive containing the HPLC stability results as browsable html pages. (ZIP) [file pone.0164577.s002.zip › diazoxide_html_results/diazoxide_bottle.html]

Stability Study Cruncher


### Preparation: bulk-oralmix, Lot: a, Condition: bottle-5

| Days | Assay (mg/mL) | | | % of initial | | | n |  |
| --- | --- | --- | --- | --- | --- | --- | --- | --- |
| 0 | 10.76 | ± | 0.05 |  |  |  | 3 | time zero |
| 7 | 10.31 | ± | 0.50 | 95.8 | ± | 4.6 | 3 | time point |
| 14 | 10.56 | ± | 0.32 | 98.1 | ± | 3.0 | 3 | time point |
| 30 | 10.55 | ± | 0.36 | 98.0 | ± | 3.4 | 3 | time point |
| 45 | 10.20 | ± | 0.32 | 94.8 | ± | 2.9 | 3 | time point |
| 60 | 10.83 | ± | 0.89 | 100.6 | ± | 8.3 | 3 | time point |
| 75 | 10.73 | ± | 0.39 | 99.7 | ± | 3.6 | 3 | time point |
| 90 | 10.31 | ± | 0.07 | 95.8 | ± | 0.6 | 3 | time point |

### Preparation: tablet-oralmix, Lot: a, Condition: bottle-5

| Days | Assay (mg/mL) | | | % of initial | | | n |  |
| --- | --- | --- | --- | --- | --- | --- | --- | --- |
| 0 | 10.19 | ± | 0.06 |  |  |  | 3 | time zero |
| 7 | 10.36 | ± | 0.04 | 101.7 | ± | 0.4 | 3 | time point |
| 14 | 10.33 | ± | 0.15 | 101.4 | ± | 1.5 | 3 | time point |
| 30 | 10.09 | ± | 0.23 | 99.0 | ± | 2.2 | 3 | time point |
| 45 | 10.23 | ± | 0.07 | 100.4 | ± | 0.7 | 3 | time point |
| 60 | 10.05 | ± | 0.02 | 98.6 | ± | 0.2 | 3 | time point |
| 75 | 10.44 | ± | 0.32 | 102.4 | ± | 3.1 | 3 | time point |
| 90 | 10.53 | ± | 0.47 | 103.4 | ± | 4.6 | 3 | time point |

### Preparation: bulk-oralmix, Lot: a, Condition: bottle-25

| Days | Assay (mg/mL) | | | % of initial | | | n |  |
| --- | --- | --- | --- | --- | --- | --- | --- | --- |
| 0 | 10.76 | ± | 0.05 |  |  |  | 3 | time zero |
| 7 | 10.74 | ± | 0.81 | 99.8 | ± | 7.5 | 3 | time point |
| 14 | 11.12 | ± | 0.44 | 103.4 | ± | 4.1 | 3 | time point |
| 30 | 10.74 | ± | 0.82 | 99.8 | ± | 7.6 | 3 | time point |
| 45 | 10.94 | ± | 0.84 | 101.7 | ± | 7.8 | 3 | time point |
| 60 | 11.34 | ± | 0.74 | 105.4 | ± | 6.8 | 3 | time point |
| 75 | 11.12 | ± | 0.74 | 103.3 | ± | 6.9 | 3 | time point |
| 90 | 11.25 | ± | 0.55 | 104.5 | ± | 5.1 | 3 | time point |

### Preparation: tablet-oralmix, Lot: a, Condition: bottle-25

| Days | Assay (mg/mL) | | | % of initial | | | n |  |
| --- | --- | --- | --- | --- | --- | --- | --- | --- |
| 0 | 10.19 | ± | 0.06 |  |  |  | 3 | time zero |
| 7 | 10.12 | ± | 0.48 | 99.4 | ± | 4.7 | 3 | time point |
| 14 | 9.85 | ± | 0.09 | 96.7 | ± | 0.9 | 3 | time point |
| 30 | 10.26 | ± | 0.55 | 100.7 | ± | 5.4 | 3 | time point |
| 45 | 9.94 | ± | 0.12 | 97.6 | ± | 1.2 | 3 | time point |
| 60 | 9.84 | ± | 0.17 | 96.6 | ± | 1.7 | 3 | time point |
| 75 | 10.11 | ± | 0.23 | 99.2 | ± | 2.3 | 3 | time point |
| 90 | 9.97 | ± | 0.20 | 97.9 | ± | 1.9 | 3 | time point |

### Preparation: bulk-oralmixsf, Lot: a, Condition: bottle-5

| Days | Assay (mg/mL) | | | % of initial | | | n |  |
| --- | --- | --- | --- | --- | --- | --- | --- | --- |
| 0 | 10.04 | ± | 0.18 |  |  |  | 3 | time zero |
| 7 | 10.21 | ± | 0.09 | 101.6 | ± | 0.9 | 3 | time point |
| 14 | 10.37 | ± | 0.42 | 103.2 | ± | 4.2 | 3 | time point |
| 30 | 10.67 | ± | 0.09 | 106.2 | ± | 0.9 | 3 | time point |
| 45 | 10.21 | ± | 0.18 | 101.6 | ± | 1.8 | 3 | time point |
| 60 | 10.53 | ± | 0.34 | 104.8 | ± | 3.4 | 3 | time point |
| 75 | 10.49 | ± | 0.35 | 104.4 | ± | 3.5 | 3 | time point |
| 90 | 10.58 | ± | 0.34 | 105.3 | ± | 3.4 | 3 | time point |

### Preparation: tablet-oralmixsf, Lot: a, Condition: bottle-5

| Days | Assay (mg/mL) | | | % of initial | | | n |  |
| --- | --- | --- | --- | --- | --- | --- | --- | --- |
| 0 | 10.22 | ± | 0.17 |  |  |  | 3 | time zero |
| 7 | 10.05 | ± | 0.38 | 98.3 | ± | 3.7 | 3 | time point |
| 14 | 9.94 | ± | 0.26 | 97.3 | ± | 2.5 | 3 | time point |
| 30 | 10.38 | ± | 0.33 | 101.5 | ± | 3.2 | 3 | time point |
| 45 | 10.61 | ± | 0.73 | 103.8 | ± | 7.1 | 3 | time point |
| 60 | 10.41 | ± | 0.21 | 101.8 | ± | 2.1 | 3 | time point |
| 75 | 10.37 | ± | 0.44 | 101.4 | ± | 4.3 | 3 | time point |
| 90 | 10.07 | ± | 0.33 | 98.6 | ± | 3.2 | 3 | time point |

### Preparation: bulk-oralmixsf, Lot: a, Condition: bottle-25

| Days | Assay (mg/mL) | | | % of initial | | | n |  |
| --- | --- | --- | --- | --- | --- | --- | --- | --- |
| 0 | 10.04 | ± | 0.18 |  |  |  | 3 | time zero |
| 7 | 10.10 | ± | 0.52 | 100.5 | ± | 5.2 | 3 | time point |
| 14 | 10.19 | ± | 0.35 | 101.5 | ± | 3.5 | 3 | time point |
| 30 | 10.50 | ± | 0.12 | 104.6 | ± | 1.2 | 3 | time point |
| 45 | 9.99 | ± | 0.38 | 99.5 | ± | 3.8 | 3 | time point |
| 60 | 10.61 | ± | 0.25 | 105.7 | ± | 2.5 | 3 | time point |
| 75 | 10.41 | ± | 0.32 | 103.7 | ± | 3.2 | 3 | time point |
| 90 | 10.18 | ± | 0.23 | 101.4 | ± | 2.3 | 3 | time point |

### Preparation: tablet-oralmixsf, Lot: a, Condition: bottle-25

| Days | Assay (mg/mL) | | | % of initial | | | n |  |
| --- | --- | --- | --- | --- | --- | --- | --- | --- |
| 0 | 10.22 | ± | 0.17 |  |  |  | 3 | time zero |
| 7 | 10.24 | ± | 0.35 | 100.2 | ± | 3.4 | 3 | time point |
| 14 | 9.81 | ± | 0.31 | 95.9 | ± | 3.0 | 3 | time point |
| 30 | 10.14 | ± | 0.42 | 99.3 | ± | 4.2 | 3 | time point |
| 45 | 10.35 | ± | 0.20 | 101.3 | ± | 2.0 | 3 | time point |
| 60 | 10.47 | ± | 0.91 | 102.5 | ± | 8.9 | 3 | time point |
| 75 | 10.89 | ± | 0.19 | 106.6 | ± | 1.8 | 3 | time point |
| 90 | 10.48 | ± | 0.16 | 102.6 | ± | 1.6 | 3 | time point |
