# Supplementary material for: Stability of Diazoxide in Extemporaneously Compounded Oral Suspensions
Source: PLoS One. 2016 Oct 11;11(10):e0164577. doi: 10.1371/journal.pone.0164577 (PMC5058506; doi:10.1371/journal.pone.0164577)
Supplement: S2 Appendix — Archive containing the HPLC stability results as browsable html pages. (ZIP) [file pone.0164577.s002.zip › diazoxide_html_results/diazoxide_bottle/index.html?preparation=bulk-oralmix&lot=a&condition=bottle-5&time=60.html]

Stability Study Cruncher


### Preparation: bulk-oralmix, Lot: a, Condition: bottle-5, Time: 60

Assay (mg/mL): 10.83 ± 0.89 (n = 3);
Assay (%TZ): 100.6 ± 8.3 (n = 3).

| Input String | Area | Cal Id | Cal Slope | Assay | Assay TZ | Assay %TZ |  |
| --- | --- | --- | --- | --- | --- | --- | --- |
| diazoxide\_bulk-oralmix\_a\_bottle-5\_60;4017744;;cal14om210;stability | 4017744 | cal14om210 | 358223 | 11.22 | 10.76 | 104.2 | calibration, time zero |
| diazoxide\_bulk-oralmix\_a\_bottle-5\_60;3513079;;cal14om210;stability | 3513079 | cal14om210 | 358223 | 9.81 | 10.76 | 91.1 | calibration, time zero |
| diazoxide\_bulk-oralmix\_a\_bottle-5\_60;4104734;;cal14om210;stability | 4104734 | cal14om210 | 358223 | 11.46 | 10.76 | 106.5 | calibration, time zero |
