# Supplementary material for: Stability of Diazoxide in Extemporaneously Compounded Oral Suspensions
Source: PLoS One. 2016 Oct 11;11(10):e0164577. doi: 10.1371/journal.pone.0164577 (PMC5058506; doi:10.1371/journal.pone.0164577)
Supplement: S2 Appendix — Archive containing the HPLC stability results as browsable html pages. (ZIP) [file pone.0164577.s002.zip › diazoxide_html_results/diazoxide_bottle/index.html?preparation=bulk-oralmix&lot=a&condition=bottle-5&time=75.html]

Stability Study Cruncher


### Preparation: bulk-oralmix, Lot: a, Condition: bottle-5, Time: 75

Assay (mg/mL): 10.73 ± 0.39 (n = 3);
Assay (%TZ): 99.7 ± 3.6 (n = 3).

| Input String | Area | Cal Id | Cal Slope | Assay | Assay TZ | Assay %TZ |  |
| --- | --- | --- | --- | --- | --- | --- | --- |
| diazoxide\_bulk-oralmix\_a\_bottle-5\_75;3819486;;cal75om210;stability | 3819486 | cal75om210 | 358017 | 10.67 | 10.76 | 99.1 | calibration, time zero |
| diazoxide\_bulk-oralmix\_a\_bottle-5\_75;3990093;;cal75om210;stability | 3990093 | cal75om210 | 358017 | 11.14 | 10.76 | 103.6 | calibration, time zero |
| diazoxide\_bulk-oralmix\_a\_bottle-5\_75;3714427;;cal75om210;stability | 3714427 | cal75om210 | 358017 | 10.37 | 10.76 | 96.4 | calibration, time zero |
