# Supplementary material for: Stability of Diazoxide in Extemporaneously Compounded Oral Suspensions
Source: PLoS One. 2016 Oct 11;11(10):e0164577. doi: 10.1371/journal.pone.0164577 (PMC5058506; doi:10.1371/journal.pone.0164577)
Supplement: S2 Appendix — Archive containing the HPLC stability results as browsable html pages. (ZIP) [file pone.0164577.s002.zip › diazoxide_html_results/diazoxide_bottle/index.html?preparation=bulk-oralmix&lot=a&condition=bottle-5&time=90.html]

Stability Study Cruncher


### Preparation: bulk-oralmix, Lot: a, Condition: bottle-5, Time: 90

Assay (mg/mL): 10.31 ± 0.07 (n = 3);
Assay (%TZ): 95.8 ± 0.6 (n = 3).

| Input String | Area | Cal Id | Cal Slope | Assay | Assay TZ | Assay %TZ |  |
| --- | --- | --- | --- | --- | --- | --- | --- |
| diazoxide\_bulk-oralmix\_a\_bottle-5\_90;3708333;;cal75om210;stability | 3708333 | cal75om210 | 358017 | 10.36 | 10.76 | 96.2 | calibration, time zero |
| diazoxide\_bulk-oralmix\_a\_bottle-5\_90;3704056;;cal75om210;stability | 3704056 | cal75om210 | 358017 | 10.35 | 10.76 | 96.1 | calibration, time zero |
| diazoxide\_bulk-oralmix\_a\_bottle-5\_90;3663319;;cal75om210;stability | 3663319 | cal75om210 | 358017 | 10.23 | 10.76 | 95.1 | calibration, time zero |
