# Supplementary material for: Stability of Diazoxide in Extemporaneously Compounded Oral Suspensions
Source: PLoS One. 2016 Oct 11;11(10):e0164577. doi: 10.1371/journal.pone.0164577 (PMC5058506; doi:10.1371/journal.pone.0164577)
Supplement: S2 Appendix — Archive containing the HPLC stability results as browsable html pages. (ZIP) [file pone.0164577.s002.zip › diazoxide_html_results/diazoxide_bottle/index.html?preparation=tablet-oralmix&lot=a&condition=bottle-5&time=7.html]

Stability Study Cruncher


### Preparation: tablet-oralmix, Lot: a, Condition: bottle-5, Time: 7

Assay (mg/mL): 10.36 ± 0.04 (n = 3);
Assay (%TZ): 101.7 ± 0.4 (n = 3).

| Input String | Area | Cal Id | Cal Slope | Assay | Assay TZ | Assay %TZ |  |
| --- | --- | --- | --- | --- | --- | --- | --- |
| diazoxide\_tablet-oralmix\_a\_bottle-5\_7;3885440;;cal7om200;stability | 3885440 | cal7om200 | 373935 | 10.39 | 10.19 | 102.0 | calibration, time zero |
| diazoxide\_tablet-oralmix\_a\_bottle-5\_7;3880581;;cal7om200;stability | 3880581 | cal7om200 | 373935 | 10.38 | 10.19 | 101.9 | calibration, time zero |
| diazoxide\_tablet-oralmix\_a\_bottle-5\_7;3860236;;cal7om200;stability | 3860236 | cal7om200 | 373935 | 10.32 | 10.19 | 101.3 | calibration, time zero |
