# Supplementary material for: Stability of Diazoxide in Extemporaneously Compounded Oral Suspensions
Source: PLoS One. 2016 Oct 11;11(10):e0164577. doi: 10.1371/journal.pone.0164577 (PMC5058506; doi:10.1371/journal.pone.0164577)
Supplement: S2 Appendix — Archive containing the HPLC stability results as browsable html pages. (ZIP) [file pone.0164577.s002.zip › diazoxide_html_results/diazoxide_bottle/index.html?preparation=tablet-oralmix&lot=a&condition=bottle-5&time=30.html]

Stability Study Cruncher


### Preparation: tablet-oralmix, Lot: a, Condition: bottle-5, Time: 30

Assay (mg/mL): 10.09 ± 0.23 (n = 3);
Assay (%TZ): 99.0 ± 2.2 (n = 3).

| Input String | Area | Cal Id | Cal Slope | Assay | Assay TZ | Assay %TZ |  |
| --- | --- | --- | --- | --- | --- | --- | --- |
| diazoxide\_tablet-oralmix\_a\_bottle-5\_30;3707182;;cal14om210;stability | 3707182 | cal14om210 | 358223 | 10.35 | 10.19 | 101.6 | calibration, time zero |
| diazoxide\_tablet-oralmix\_a\_bottle-5\_30;3560216;;cal14om210;stability | 3560216 | cal14om210 | 358223 | 9.94 | 10.19 | 97.6 | calibration, time zero |
| diazoxide\_tablet-oralmix\_a\_bottle-5\_30;3575291;;cal14om210;stability | 3575291 | cal14om210 | 358223 | 9.98 | 10.19 | 98.0 | calibration, time zero |
