# Supplementary material for: Stability of Diazoxide in Extemporaneously Compounded Oral Suspensions
Source: PLoS One. 2016 Oct 11;11(10):e0164577. doi: 10.1371/journal.pone.0164577 (PMC5058506; doi:10.1371/journal.pone.0164577)
Supplement: S2 Appendix — Archive containing the HPLC stability results as browsable html pages. (ZIP) [file pone.0164577.s002.zip › diazoxide_html_results/diazoxide_bottle/index.html?preparation=tablet-oralmix&lot=a&condition=bottle-5&time=45.html]

Stability Study Cruncher


### Preparation: tablet-oralmix, Lot: a, Condition: bottle-5, Time: 45

Assay (mg/mL): 10.23 ± 0.07 (n = 3);
Assay (%TZ): 100.4 ± 0.7 (n = 3).

| Input String | Area | Cal Id | Cal Slope | Assay | Assay TZ | Assay %TZ |  |
| --- | --- | --- | --- | --- | --- | --- | --- |
| diazoxide\_tablet-oralmix\_a\_bottle-5\_45;3691138;;cal14om210;stability | 3691138 | cal14om210 | 358223 | 10.30 | 10.19 | 101.1 | calibration, time zero |
| diazoxide\_tablet-oralmix\_a\_bottle-5\_45;3640202;;cal14om210;stability | 3640202 | cal14om210 | 358223 | 10.16 | 10.19 | 99.8 | calibration, time zero |
| diazoxide\_tablet-oralmix\_a\_bottle-5\_45;3663260;;cal14om210;stability | 3663260 | cal14om210 | 358223 | 10.23 | 10.19 | 100.4 | calibration, time zero |
