# Supplementary material for: Stability of Diazoxide in Extemporaneously Compounded Oral Suspensions
Source: PLoS One. 2016 Oct 11;11(10):e0164577. doi: 10.1371/journal.pone.0164577 (PMC5058506; doi:10.1371/journal.pone.0164577)
Supplement: S2 Appendix — Archive containing the HPLC stability results as browsable html pages. (ZIP) [file pone.0164577.s002.zip › diazoxide_html_results/diazoxide_bottle/index.html?preparation=bulk-oralmix&lot=a&condition=bottle-25&time=7.html]

Stability Study Cruncher


### Preparation: bulk-oralmix, Lot: a, Condition: bottle-25, Time: 7

Assay (mg/mL): 10.74 ± 0.81 (n = 3);
Assay (%TZ): 99.8 ± 7.5 (n = 3).

| Input String | Area | Cal Id | Cal Slope | Assay | Assay TZ | Assay %TZ |  |
| --- | --- | --- | --- | --- | --- | --- | --- |
| diazoxide\_bulk-oralmix\_a\_bottle-25\_7;4347725;;cal7om200;stability | 4347725 | cal7om200 | 373935 | 11.63 | 10.76 | 108.0 | calibration, time zero |
| diazoxide\_bulk-oralmix\_a\_bottle-25\_7;3753170;;cal7om200;stability | 3753170 | cal7om200 | 373935 | 10.04 | 10.76 | 93.3 | calibration, time zero |
| diazoxide\_bulk-oralmix\_a\_bottle-25\_7;3761602;;cal7om210;stability | 3761602 | cal7om210 | 356128 | 10.56 | 10.76 | 98.1 | calibration, time zero |
