# Supplementary material for: Stability of Diazoxide in Extemporaneously Compounded Oral Suspensions
Source: PLoS One. 2016 Oct 11;11(10):e0164577. doi: 10.1371/journal.pone.0164577 (PMC5058506; doi:10.1371/journal.pone.0164577)
Supplement: S2 Appendix — Archive containing the HPLC stability results as browsable html pages. (ZIP) [file pone.0164577.s002.zip › diazoxide_html_results/diazoxide_bottle/index.html?preparation=bulk-oralmix&lot=a&condition=bottle-25&time=14.html]

Stability Study Cruncher


### Preparation: bulk-oralmix, Lot: a, Condition: bottle-25, Time: 14

Assay (mg/mL): 11.12 ± 0.44 (n = 3);
Assay (%TZ): 103.4 ± 4.1 (n = 3).

| Input String | Area | Cal Id | Cal Slope | Assay | Assay TZ | Assay %TZ |  |
| --- | --- | --- | --- | --- | --- | --- | --- |
| diazoxide\_bulk-oralmix\_a\_bottle-25\_14;4004077;;cal14om210;stability | 4004077 | cal14om210 | 358223 | 11.18 | 10.76 | 103.9 | calibration, time zero |
| diazoxide\_bulk-oralmix\_a\_bottle-25\_14;4133087;;cal14om210;stability | 4133087 | cal14om210 | 358223 | 11.54 | 10.76 | 107.2 | calibration, time zero |
| diazoxide\_bulk-oralmix\_a\_bottle-25\_14;3816657;;cal14om210;stability | 3816657 | cal14om210 | 358223 | 10.65 | 10.76 | 99.0 | calibration, time zero |
