# Supplementary material for: Stability of Diazoxide in Extemporaneously Compounded Oral Suspensions
Source: PLoS One. 2016 Oct 11;11(10):e0164577. doi: 10.1371/journal.pone.0164577 (PMC5058506; doi:10.1371/journal.pone.0164577)
Supplement: S2 Appendix — Archive containing the HPLC stability results as browsable html pages. (ZIP) [file pone.0164577.s002.zip › diazoxide_html_results/diazoxide_bottle/index.html?preparation=bulk-oralmix&lot=a&condition=bottle-25&time=30.html]

Stability Study Cruncher


### Preparation: bulk-oralmix, Lot: a, Condition: bottle-25, Time: 30

Assay (mg/mL): 10.74 ± 0.82 (n = 3);
Assay (%TZ): 99.8 ± 7.6 (n = 3).

| Input String | Area | Cal Id | Cal Slope | Assay | Assay TZ | Assay %TZ |  |
| --- | --- | --- | --- | --- | --- | --- | --- |
| diazoxide\_bulk-oralmix\_a\_bottle-25\_30;4157984;;cal14om210;stability | 4157984 | cal14om210 | 358223 | 11.61 | 10.76 | 107.8 | calibration, time zero |
| diazoxide\_bulk-oralmix\_a\_bottle-25\_30;3811777;;cal14om210;stability | 3811777 | cal14om210 | 358223 | 10.64 | 10.76 | 98.9 | calibration, time zero |
| diazoxide\_bulk-oralmix\_a\_bottle-25\_30;3577408;;cal14om210;stability | 3577408 | cal14om210 | 358223 | 9.99 | 10.76 | 92.8 | calibration, time zero |
