# Supplementary material for: Stability of Diazoxide in Extemporaneously Compounded Oral Suspensions
Source: PLoS One. 2016 Oct 11;11(10):e0164577. doi: 10.1371/journal.pone.0164577 (PMC5058506; doi:10.1371/journal.pone.0164577)
Supplement: S2 Appendix — Archive containing the HPLC stability results as browsable html pages. (ZIP) [file pone.0164577.s002.zip › diazoxide_html_results/diazoxide_bottle/index.html?preparation=bulk-oralmix&lot=a&condition=bottle-25&time=90.html]

Stability Study Cruncher


### Preparation: bulk-oralmix, Lot: a, Condition: bottle-25, Time: 90

Assay (mg/mL): 11.25 ± 0.55 (n = 3);
Assay (%TZ): 104.5 ± 5.1 (n = 3).

| Input String | Area | Cal Id | Cal Slope | Assay | Assay TZ | Assay %TZ |  |
| --- | --- | --- | --- | --- | --- | --- | --- |
| diazoxide\_bulk-oralmix\_a\_bottle-25\_90;4070280;;cal75om210;stability | 4070280 | cal75om210 | 358017 | 11.37 | 10.76 | 105.6 | calibration, time zero |
| diazoxide\_bulk-oralmix\_a\_bottle-25\_90;4197962;;cal75om210;stability | 4197962 | cal75om210 | 358017 | 11.73 | 10.76 | 108.9 | calibration, time zero |
| diazoxide\_bulk-oralmix\_a\_bottle-25\_90;3814679;;cal75om210;stability | 3814679 | cal75om210 | 358017 | 10.66 | 10.76 | 99.0 | calibration, time zero |
