# Supplementary material for: Stability of Diazoxide in Extemporaneously Compounded Oral Suspensions
Source: PLoS One. 2016 Oct 11;11(10):e0164577. doi: 10.1371/journal.pone.0164577 (PMC5058506; doi:10.1371/journal.pone.0164577)
Supplement: S2 Appendix — Archive containing the HPLC stability results as browsable html pages. (ZIP) [file pone.0164577.s002.zip › diazoxide_html_results/diazoxide_bottle/index.html?preparation=tablet-oralmix&lot=a&condition=bottle-25&time=7.html]

Stability Study Cruncher


### Preparation: tablet-oralmix, Lot: a, Condition: bottle-25, Time: 7

Assay (mg/mL): 10.12 ± 0.48 (n = 3);
Assay (%TZ): 99.4 ± 4.7 (n = 3).

| Input String | Area | Cal Id | Cal Slope | Assay | Assay TZ | Assay %TZ |  |
| --- | --- | --- | --- | --- | --- | --- | --- |
| diazoxide\_tablet-oralmix\_a\_bottle-25\_7;3693750;;cal7om200;stability | 3693750 | cal7om200 | 373935 | 9.88 | 10.19 | 97.0 | calibration, time zero |
| diazoxide\_tablet-oralmix\_a\_bottle-25\_7;3671173;;cal7om200;stability | 3671173 | cal7om200 | 373935 | 9.82 | 10.19 | 96.4 | calibration, time zero |
| diazoxide\_tablet-oralmix\_a\_bottle-25\_7;3989996;;cal7om200;stability | 3989996 | cal7om200 | 373935 | 10.67 | 10.19 | 104.7 | calibration, time zero |
