# Supplementary material for: Stability of Diazoxide in Extemporaneously Compounded Oral Suspensions
Source: PLoS One. 2016 Oct 11;11(10):e0164577. doi: 10.1371/journal.pone.0164577 (PMC5058506; doi:10.1371/journal.pone.0164577)
Supplement: S2 Appendix — Archive containing the HPLC stability results as browsable html pages. (ZIP) [file pone.0164577.s002.zip › diazoxide_html_results/diazoxide_bottle/index.html?preparation=tablet-oralmix&lot=a&condition=bottle-25&time=45.html]

Stability Study Cruncher


### Preparation: tablet-oralmix, Lot: a, Condition: bottle-25, Time: 45

Assay (mg/mL): 9.94 ± 0.12 (n = 3);
Assay (%TZ): 97.6 ± 1.2 (n = 3).

| Input String | Area | Cal Id | Cal Slope | Assay | Assay TZ | Assay %TZ |  |
| --- | --- | --- | --- | --- | --- | --- | --- |
| diazoxide\_tablet-oralmix\_a\_bottle-25\_45;3592078;;cal14om210;stability | 3592078 | cal14om210 | 358223 | 10.03 | 10.19 | 98.4 | calibration, time zero |
| diazoxide\_tablet-oralmix\_a\_bottle-25\_45;3511119;;cal14om210;stability | 3511119 | cal14om210 | 358223 | 9.80 | 10.19 | 96.2 | calibration, time zero |
| diazoxide\_tablet-oralmix\_a\_bottle-25\_45;3582010;;cal14om210;stability | 3582010 | cal14om210 | 358223 | 10.00 | 10.19 | 98.2 | calibration, time zero |
