# Supplementary material for: Stability of Diazoxide in Extemporaneously Compounded Oral Suspensions
Source: PLoS One. 2016 Oct 11;11(10):e0164577. doi: 10.1371/journal.pone.0164577 (PMC5058506; doi:10.1371/journal.pone.0164577)
Supplement: S2 Appendix — Archive containing the HPLC stability results as browsable html pages. (ZIP) [file pone.0164577.s002.zip › diazoxide_html_results/diazoxide_bottle/index.html?preparation=tablet-oralmix&lot=a&condition=bottle-25&time=60.html]

Stability Study Cruncher


### Preparation: tablet-oralmix, Lot: a, Condition: bottle-25, Time: 60

Assay (mg/mL): 9.84 ± 0.17 (n = 3);
Assay (%TZ): 96.6 ± 1.7 (n = 3).

| Input String | Area | Cal Id | Cal Slope | Assay | Assay TZ | Assay %TZ |  |
| --- | --- | --- | --- | --- | --- | --- | --- |
| diazoxide\_tablet-oralmix\_a\_bottle-25\_60;3572229;;cal14om210;stability | 3572229 | cal14om210 | 358223 | 9.97 | 10.19 | 97.9 | calibration, time zero |
| diazoxide\_tablet-oralmix\_a\_bottle-25\_60;3457447;;cal14om210;stability | 3457447 | cal14om210 | 358223 | 9.65 | 10.19 | 94.7 | calibration, time zero |
| diazoxide\_tablet-oralmix\_a\_bottle-25\_60;3546742;;cal14om210;stability | 3546742 | cal14om210 | 358223 | 9.90 | 10.19 | 97.2 | calibration, time zero |
