# Supplementary material for: Stability of Diazoxide in Extemporaneously Compounded Oral Suspensions
Source: PLoS One. 2016 Oct 11;11(10):e0164577. doi: 10.1371/journal.pone.0164577 (PMC5058506; doi:10.1371/journal.pone.0164577)
Supplement: S2 Appendix — Archive containing the HPLC stability results as browsable html pages. (ZIP) [file pone.0164577.s002.zip › diazoxide_html_results/diazoxide_bottle/index.html?preparation=tablet-oralmix&lot=a&condition=bottle-25&time=75.html]

Stability Study Cruncher


### Preparation: tablet-oralmix, Lot: a, Condition: bottle-25, Time: 75

Assay (mg/mL): 10.11 ± 0.23 (n = 3);
Assay (%TZ): 99.2 ± 2.3 (n = 3).

| Input String | Area | Cal Id | Cal Slope | Assay | Assay TZ | Assay %TZ |  |
| --- | --- | --- | --- | --- | --- | --- | --- |
| diazoxide\_tablet-oralmix\_a\_bottle-25\_75;3571966;;cal75om210;stability | 3571966 | cal75om210 | 358017 | 9.98 | 10.19 | 97.9 | calibration, time zero |
| diazoxide\_tablet-oralmix\_a\_bottle-25\_75;3570842;;cal75om210;stability | 3570842 | cal75om210 | 358017 | 9.97 | 10.19 | 97.9 | calibration, time zero |
| diazoxide\_tablet-oralmix\_a\_bottle-25\_75;3714486;;cal75om210;stability | 3714486 | cal75om210 | 358017 | 10.38 | 10.19 | 101.8 | calibration, time zero |
