# Supplementary material for: Stability of Diazoxide in Extemporaneously Compounded Oral Suspensions
Source: PLoS One. 2016 Oct 11;11(10):e0164577. doi: 10.1371/journal.pone.0164577 (PMC5058506; doi:10.1371/journal.pone.0164577)
Supplement: S2 Appendix — Archive containing the HPLC stability results as browsable html pages. (ZIP) [file pone.0164577.s002.zip › diazoxide_html_results/diazoxide_bottle/index.html?preparation=bulk-oralmixsf&lot=a.html]

Stability Study Cruncher


### Preparation: bulk-oralmixsf, Lot: a

Assay: 10.04 ± 0.18 mg/mL (n = 3).

| Input String | Area | Cal Id | Cal Slope | Assay |  |
| --- | --- | --- | --- | --- | --- |
| diazoxide\_bulk-oralmixsf\_a;3722999;;calt0sf200;time zero | 3722999 | calt0sf200 | 374038 | 9.95 | calibration |
| diazoxide\_bulk-oralmixsf\_a;3713132;;calt0sf200;time zero | 3713132 | calt0sf200 | 374038 | 9.93 | calibration |
| diazoxide\_bulk-oralmixsf\_a;3651923;;calt0sf210;time zero | 3651923 | calt0sf210 | 356227 | 10.25 | calibration |
