# Supplementary material for: Stability of Diazoxide in Extemporaneously Compounded Oral Suspensions
Source: PLoS One. 2016 Oct 11;11(10):e0164577. doi: 10.1371/journal.pone.0164577 (PMC5058506; doi:10.1371/journal.pone.0164577)
Supplement: S2 Appendix — Archive containing the HPLC stability results as browsable html pages. (ZIP) [file pone.0164577.s002.zip › diazoxide_html_results/diazoxide_bottle/index.html?preparation=tablet-oralmix&lot=a&condition=bottle-25&time=90.html]

Stability Study Cruncher


### Preparation: tablet-oralmix, Lot: a, Condition: bottle-25, Time: 90

Assay (mg/mL): 9.97 ± 0.20 (n = 3);
Assay (%TZ): 97.9 ± 1.9 (n = 3).

| Input String | Area | Cal Id | Cal Slope | Assay | Assay TZ | Assay %TZ |  |
| --- | --- | --- | --- | --- | --- | --- | --- |
| diazoxide\_tablet-oralmix\_a\_bottle-25\_90;3652205;;cal75om210;stability | 3652205 | cal75om210 | 358017 | 10.20 | 10.19 | 100.1 | calibration, time zero |
| diazoxide\_tablet-oralmix\_a\_bottle-25\_90;3529322;;cal75om210;stability | 3529322 | cal75om210 | 358017 | 9.86 | 10.19 | 96.8 | calibration, time zero |
| diazoxide\_tablet-oralmix\_a\_bottle-25\_90;3531452;;cal75om210;stability | 3531452 | cal75om210 | 358017 | 9.86 | 10.19 | 96.8 | calibration, time zero |
