# Supplementary material for: Stability of Diazoxide in Extemporaneously Compounded Oral Suspensions
Source: PLoS One. 2016 Oct 11;11(10):e0164577. doi: 10.1371/journal.pone.0164577 (PMC5058506; doi:10.1371/journal.pone.0164577)
Supplement: S2 Appendix — Archive containing the HPLC stability results as browsable html pages. (ZIP) [file pone.0164577.s002.zip › diazoxide_html_results/diazoxide_bottle/index.html?preparation=bulk-oralmixsf&lot=a&condition=bottle-5&time=7.html]

Stability Study Cruncher


### Preparation: bulk-oralmixsf, Lot: a, Condition: bottle-5, Time: 7

Assay (mg/mL): 10.21 ± 0.09 (n = 3);
Assay (%TZ): 101.6 ± 0.9 (n = 3).

| Input String | Area | Cal Id | Cal Slope | Assay | Assay TZ | Assay %TZ |  |
| --- | --- | --- | --- | --- | --- | --- | --- |
| diazoxide\_bulk-oralmixsf\_a\_bottle-5\_7;3665641;;cal7sf210;stability | 3665641 | cal7sf210 | 355486 | 10.31 | 10.04 | 102.7 | calibration, time zero |
| diazoxide\_bulk-oralmixsf\_a\_bottle-5\_7;3802134;;cal7sf200;stability | 3802134 | cal7sf200 | 373260 | 10.19 | 10.04 | 101.4 | calibration, time zero |
| diazoxide\_bulk-oralmixsf\_a\_bottle-5\_7;3781351;;cal7sf200;stability | 3781351 | cal7sf200 | 373260 | 10.13 | 10.04 | 100.9 | calibration, time zero |
