# Supplementary material for: Stability of Diazoxide in Extemporaneously Compounded Oral Suspensions
Source: PLoS One. 2016 Oct 11;11(10):e0164577. doi: 10.1371/journal.pone.0164577 (PMC5058506; doi:10.1371/journal.pone.0164577)
Supplement: S2 Appendix — Archive containing the HPLC stability results as browsable html pages. (ZIP) [file pone.0164577.s002.zip › diazoxide_html_results/diazoxide_bottle/index.html?preparation=bulk-oralmixsf&lot=a&condition=bottle-5&time=14.html]

Stability Study Cruncher


### Preparation: bulk-oralmixsf, Lot: a, Condition: bottle-5, Time: 14

Assay (mg/mL): 10.37 ± 0.42 (n = 3);
Assay (%TZ): 103.2 ± 4.2 (n = 3).

| Input String | Area | Cal Id | Cal Slope | Assay | Assay TZ | Assay %TZ |  |
| --- | --- | --- | --- | --- | --- | --- | --- |
| diazoxide\_bulk-oralmixsf\_a\_bottle-5\_14;3626872;;cal14sf210;stability | 3626872 | cal14sf210 | 359483 | 10.09 | 10.04 | 100.4 | calibration, time zero |
| diazoxide\_bulk-oralmixsf\_a\_bottle-5\_14;3902623;;cal14sf210;stability | 3902623 | cal14sf210 | 359483 | 10.86 | 10.04 | 108.1 | calibration, time zero |
| diazoxide\_bulk-oralmixsf\_a\_bottle-5\_14;3652547;;cal14sf210;stability | 3652547 | cal14sf210 | 359483 | 10.16 | 10.04 | 101.2 | calibration, time zero |
