# Supplementary material for: Stability of Diazoxide in Extemporaneously Compounded Oral Suspensions
Source: PLoS One. 2016 Oct 11;11(10):e0164577. doi: 10.1371/journal.pone.0164577 (PMC5058506; doi:10.1371/journal.pone.0164577)
Supplement: S2 Appendix — Archive containing the HPLC stability results as browsable html pages. (ZIP) [file pone.0164577.s002.zip › diazoxide_html_results/diazoxide_bottle/index.html?preparation=bulk-oralmixsf&lot=a&condition=bottle-5&time=75.html]

Stability Study Cruncher


### Preparation: bulk-oralmixsf, Lot: a, Condition: bottle-5, Time: 75

Assay (mg/mL): 10.49 ± 0.35 (n = 3);
Assay (%TZ): 104.4 ± 3.5 (n = 3).

| Input String | Area | Cal Id | Cal Slope | Assay | Assay TZ | Assay %TZ |  |
| --- | --- | --- | --- | --- | --- | --- | --- |
| diazoxide\_bulk-oralmixsf\_a\_bottle-5\_75;3899483;;cal60sf210;stability | 3899483 | cal60sf210 | 358176 | 10.89 | 10.04 | 108.4 | calibration, time zero |
| diazoxide\_bulk-oralmixsf\_a\_bottle-5\_75;3699838;;cal60sf210;stability | 3699838 | cal60sf210 | 358176 | 10.33 | 10.04 | 102.8 | calibration, time zero |
| diazoxide\_bulk-oralmixsf\_a\_bottle-5\_75;3668444;;cal60sf210;stability | 3668444 | cal60sf210 | 358176 | 10.24 | 10.04 | 102.0 | calibration, time zero |
