# Supplementary material for: Stability of Diazoxide in Extemporaneously Compounded Oral Suspensions
Source: PLoS One. 2016 Oct 11;11(10):e0164577. doi: 10.1371/journal.pone.0164577 (PMC5058506; doi:10.1371/journal.pone.0164577)
Supplement: S2 Appendix — Archive containing the HPLC stability results as browsable html pages. (ZIP) [file pone.0164577.s002.zip › diazoxide_html_results/diazoxide_bottle/index.html?preparation=tablet-oralmixsf&lot=a.html]

Stability Study Cruncher


### Preparation: tablet-oralmixsf, Lot: a

Assay: 10.22 ± 0.17 mg/mL (n = 3).

| Input String | Area | Cal Id | Cal Slope | Assay |  |
| --- | --- | --- | --- | --- | --- |
| diazoxide\_tablet-oralmixsf\_a;3812396;;calt0sf200;time zero | 3812396 | calt0sf200 | 374038 | 10.19 | calibration |
| diazoxide\_tablet-oralmixsf\_a;3889843;;calt0sf200;time zero | 3889843 | calt0sf200 | 374038 | 10.40 | calibration |
| diazoxide\_tablet-oralmixsf\_a;3766297;;calt0sf200;time zero | 3766297 | calt0sf200 | 374038 | 10.07 | calibration |
