# Supplementary material for: Stability of Diazoxide in Extemporaneously Compounded Oral Suspensions
Source: PLoS One. 2016 Oct 11;11(10):e0164577. doi: 10.1371/journal.pone.0164577 (PMC5058506; doi:10.1371/journal.pone.0164577)
Supplement: S2 Appendix — Archive containing the HPLC stability results as browsable html pages. (ZIP) [file pone.0164577.s002.zip › diazoxide_html_results/diazoxide_bottle/index.html?preparation=tablet-oralmixsf&lot=a&condition=bottle-5&time=7.html]

Stability Study Cruncher


### Preparation: tablet-oralmixsf, Lot: a, Condition: bottle-5, Time: 7

Assay (mg/mL): 10.05 ± 0.38 (n = 3);
Assay (%TZ): 98.3 ± 3.7 (n = 3).

| Input String | Area | Cal Id | Cal Slope | Assay | Assay TZ | Assay %TZ |  |
| --- | --- | --- | --- | --- | --- | --- | --- |
| diazoxide\_tablet-oralmixsf\_a\_bottle-5\_7;3903932;;cal7sf200;stability | 3903932 | cal7sf200 | 373260 | 10.46 | 10.22 | 102.3 | calibration, time zero |
| diazoxide\_tablet-oralmixsf\_a\_bottle-5\_7;3626568;;cal7sf200;stability | 3626568 | cal7sf200 | 373260 | 9.72 | 10.22 | 95.1 | calibration, time zero |
| diazoxide\_tablet-oralmixsf\_a\_bottle-5\_7;3718153;;cal7sf200;stability | 3718153 | cal7sf200 | 373260 | 9.96 | 10.22 | 97.5 | calibration, time zero |
