# Supplementary material for: Stability of Diazoxide in Extemporaneously Compounded Oral Suspensions
Source: PLoS One. 2016 Oct 11;11(10):e0164577. doi: 10.1371/journal.pone.0164577 (PMC5058506; doi:10.1371/journal.pone.0164577)
Supplement: S2 Appendix — Archive containing the HPLC stability results as browsable html pages. (ZIP) [file pone.0164577.s002.zip › diazoxide_html_results/diazoxide_bottle/index.html?preparation=tablet-oralmixsf&lot=a&condition=bottle-5&time=14.html]

Stability Study Cruncher


### Preparation: tablet-oralmixsf, Lot: a, Condition: bottle-5, Time: 14

Assay (mg/mL): 9.94 ± 0.26 (n = 3);
Assay (%TZ): 97.3 ± 2.5 (n = 3).

| Input String | Area | Cal Id | Cal Slope | Assay | Assay TZ | Assay %TZ |  |
| --- | --- | --- | --- | --- | --- | --- | --- |
| diazoxide\_tablet-oralmixsf\_a\_bottle-5\_14;3471760;;cal14sf210;stability | 3471760 | cal14sf210 | 359483 | 9.66 | 10.22 | 94.5 | calibration, time zero |
| diazoxide\_tablet-oralmixsf\_a\_bottle-5\_14;3597118;;cal14sf210;stability | 3597118 | cal14sf210 | 359483 | 10.01 | 10.22 | 97.9 | calibration, time zero |
| diazoxide\_tablet-oralmixsf\_a\_bottle-5\_14;3650841;;cal14sf210;stability | 3650841 | cal14sf210 | 359483 | 10.16 | 10.22 | 99.4 | calibration, time zero |
