# Supplementary material for: Stability of Diazoxide in Extemporaneously Compounded Oral Suspensions
Source: PLoS One. 2016 Oct 11;11(10):e0164577. doi: 10.1371/journal.pone.0164577 (PMC5058506; doi:10.1371/journal.pone.0164577)
Supplement: S2 Appendix — Archive containing the HPLC stability results as browsable html pages. (ZIP) [file pone.0164577.s002.zip › diazoxide_html_results/diazoxide_bottle/index.html?preparation=tablet-oralmixsf&lot=a&condition=bottle-5&time=75.html]

Stability Study Cruncher


### Preparation: tablet-oralmixsf, Lot: a, Condition: bottle-5, Time: 75

Assay (mg/mL): 10.37 ± 0.44 (n = 3);
Assay (%TZ): 101.4 ± 4.3 (n = 3).

| Input String | Area | Cal Id | Cal Slope | Assay | Assay TZ | Assay %TZ |  |
| --- | --- | --- | --- | --- | --- | --- | --- |
| diazoxide\_tablet-oralmixsf\_a\_bottle-5\_75;3633402;;cal60sf210;stability | 3633402 | cal60sf210 | 358176 | 10.14 | 10.22 | 99.3 | calibration, time zero |
| diazoxide\_tablet-oralmixsf\_a\_bottle-5\_75;3895436;;cal60sf210;stability | 3895436 | cal60sf210 | 358176 | 10.88 | 10.22 | 106.4 | calibration, time zero |
| diazoxide\_tablet-oralmixsf\_a\_bottle-5\_75;3609145;;cal60sf210;stability | 3609145 | cal60sf210 | 358176 | 10.08 | 10.22 | 98.6 | calibration, time zero |
