# Supplementary material for: Stability of Diazoxide in Extemporaneously Compounded Oral Suspensions
Source: PLoS One. 2016 Oct 11;11(10):e0164577. doi: 10.1371/journal.pone.0164577 (PMC5058506; doi:10.1371/journal.pone.0164577)
Supplement: S2 Appendix — Archive containing the HPLC stability results as browsable html pages. (ZIP) [file pone.0164577.s002.zip › diazoxide_html_results/diazoxide_bottle/index.html?preparation=bulk-oralmixsf&lot=a&condition=bottle-25&time=7.html]

Stability Study Cruncher


### Preparation: bulk-oralmixsf, Lot: a, Condition: bottle-25, Time: 7

Assay (mg/mL): 10.10 ± 0.52 (n = 3);
Assay (%TZ): 100.5 ± 5.2 (n = 3).

| Input String | Area | Cal Id | Cal Slope | Assay | Assay TZ | Assay %TZ |  |
| --- | --- | --- | --- | --- | --- | --- | --- |
| diazoxide\_bulk-oralmixsf\_a\_bottle-25\_7;3982409;;cal7sf200;stability | 3982409 | cal7sf200 | 373260 | 10.67 | 10.04 | 106.2 | calibration, time zero |
| diazoxide\_bulk-oralmixsf\_a\_bottle-25\_7;3606375;;cal7sf200;stability | 3606375 | cal7sf200 | 373260 | 9.66 | 10.04 | 96.2 | calibration, time zero |
| diazoxide\_bulk-oralmixsf\_a\_bottle-25\_7;3717935;;cal7sf200;stability | 3717935 | cal7sf200 | 373260 | 9.96 | 10.04 | 99.2 | calibration, time zero |
