# Supplementary material for: Stability of Diazoxide in Extemporaneously Compounded Oral Suspensions
Source: PLoS One. 2016 Oct 11;11(10):e0164577. doi: 10.1371/journal.pone.0164577 (PMC5058506; doi:10.1371/journal.pone.0164577)
Supplement: S2 Appendix — Archive containing the HPLC stability results as browsable html pages. (ZIP) [file pone.0164577.s002.zip › diazoxide_html_results/diazoxide_bottle/index.html?preparation=bulk-oralmixsf&lot=a&condition=bottle-25&time=14.html]

Stability Study Cruncher


### Preparation: bulk-oralmixsf, Lot: a, Condition: bottle-25, Time: 14

Assay (mg/mL): 10.19 ± 0.35 (n = 3);
Assay (%TZ): 101.5 ± 3.5 (n = 3).

| Input String | Area | Cal Id | Cal Slope | Assay | Assay TZ | Assay %TZ |  |
| --- | --- | --- | --- | --- | --- | --- | --- |
| diazoxide\_bulk-oralmixsf\_a\_bottle-25\_14;3635782;;cal14sf210;stability | 3635782 | cal14sf210 | 359483 | 10.11 | 10.04 | 100.7 | calibration, time zero |
| diazoxide\_bulk-oralmixsf\_a\_bottle-25\_14;3555866;;cal14sf210;stability | 3555866 | cal14sf210 | 359483 | 9.89 | 10.04 | 98.5 | calibration, time zero |
| diazoxide\_bulk-oralmixsf\_a\_bottle-25\_14;3801251;;cal14sf210;stability | 3801251 | cal14sf210 | 359483 | 10.57 | 10.04 | 105.3 | calibration, time zero |
