# Supplementary material for: Stability of Diazoxide in Extemporaneously Compounded Oral Suspensions
Source: PLoS One. 2016 Oct 11;11(10):e0164577. doi: 10.1371/journal.pone.0164577 (PMC5058506; doi:10.1371/journal.pone.0164577)
Supplement: S2 Appendix — Archive containing the HPLC stability results as browsable html pages. (ZIP) [file pone.0164577.s002.zip › diazoxide_html_results/diazoxide_bottle/index.html?preparation=bulk-oralmixsf&lot=a&condition=bottle-25&time=45.html]

Stability Study Cruncher


### Preparation: bulk-oralmixsf, Lot: a, Condition: bottle-25, Time: 45

Assay (mg/mL): 9.99 ± 0.38 (n = 3);
Assay (%TZ): 99.5 ± 3.8 (n = 3).

| Input String | Area | Cal Id | Cal Slope | Assay | Assay TZ | Assay %TZ |  |
| --- | --- | --- | --- | --- | --- | --- | --- |
| diazoxide\_bulk-oralmixsf\_a\_bottle-25\_45;3584833;;cal30sf210;stability | 3584833 | cal30sf210 | 358295 | 10.01 | 10.04 | 99.6 | calibration, time zero |
| diazoxide\_bulk-oralmixsf\_a\_bottle-25\_45;3440444;;cal30sf210;stability | 3440444 | cal30sf210 | 358295 | 9.60 | 10.04 | 95.6 | calibration, time zero |
| diazoxide\_bulk-oralmixsf\_a\_bottle-25\_45;3712979;;cal30sf210;stability | 3712979 | cal30sf210 | 358295 | 10.36 | 10.04 | 103.2 | calibration, time zero |
