# Supplementary material for: Stability of Diazoxide in Extemporaneously Compounded Oral Suspensions
Source: PLoS One. 2016 Oct 11;11(10):e0164577. doi: 10.1371/journal.pone.0164577 (PMC5058506; doi:10.1371/journal.pone.0164577)
Supplement: S2 Appendix — Archive containing the HPLC stability results as browsable html pages. (ZIP) [file pone.0164577.s002.zip › diazoxide_html_results/diazoxide_bottle/index.html?preparation=bulk-oralmixsf&lot=a&condition=bottle-25&time=75.html]

Stability Study Cruncher


### Preparation: bulk-oralmixsf, Lot: a, Condition: bottle-25, Time: 75

Assay (mg/mL): 10.41 ± 0.32 (n = 3);
Assay (%TZ): 103.7 ± 3.2 (n = 3).

| Input String | Area | Cal Id | Cal Slope | Assay | Assay TZ | Assay %TZ |  |
| --- | --- | --- | --- | --- | --- | --- | --- |
| diazoxide\_bulk-oralmixsf\_a\_bottle-25\_75;3786901;;cal60sf210;stability | 3786901 | cal60sf210 | 358176 | 10.57 | 10.04 | 105.3 | calibration, time zero |
| diazoxide\_bulk-oralmixsf\_a\_bottle-25\_75;3595576;;cal60sf210;stability | 3595576 | cal60sf210 | 358176 | 10.04 | 10.04 | 99.9 | calibration, time zero |
| diazoxide\_bulk-oralmixsf\_a\_bottle-25\_75;3804291;;cal60sf210;stability | 3804291 | cal60sf210 | 358176 | 10.62 | 10.04 | 105.7 | calibration, time zero |
