# Supplementary material for: Stability of Diazoxide in Extemporaneously Compounded Oral Suspensions
Source: PLoS One. 2016 Oct 11;11(10):e0164577. doi: 10.1371/journal.pone.0164577 (PMC5058506; doi:10.1371/journal.pone.0164577)
Supplement: S2 Appendix — Archive containing the HPLC stability results as browsable html pages. (ZIP) [file pone.0164577.s002.zip › diazoxide_html_results/diazoxide_bottle/index.html?preparation=bulk-oralmixsf&lot=a&condition=bottle-25&time=90.html]

Stability Study Cruncher


### Preparation: bulk-oralmixsf, Lot: a, Condition: bottle-25, Time: 90

Assay (mg/mL): 10.18 ± 0.23 (n = 3);
Assay (%TZ): 101.4 ± 2.3 (n = 3).

| Input String | Area | Cal Id | Cal Slope | Assay | Assay TZ | Assay %TZ |  |
| --- | --- | --- | --- | --- | --- | --- | --- |
| diazoxide\_bulk-oralmixsf\_a\_bottle-25\_90;3676500;;cal60sf210;stability | 3676500 | cal60sf210 | 358176 | 10.26 | 10.04 | 102.2 | calibration, time zero |
| diazoxide\_bulk-oralmixsf\_a\_bottle-25\_90;3554251;;cal60sf210;stability | 3554251 | cal60sf210 | 358176 | 9.92 | 10.04 | 98.8 | calibration, time zero |
| diazoxide\_bulk-oralmixsf\_a\_bottle-25\_90;3711692;;cal60sf210;stability | 3711692 | cal60sf210 | 358176 | 10.36 | 10.04 | 103.2 | calibration, time zero |
