# Supplementary material for: Stability of Diazoxide in Extemporaneously Compounded Oral Suspensions
Source: PLoS One. 2016 Oct 11;11(10):e0164577. doi: 10.1371/journal.pone.0164577 (PMC5058506; doi:10.1371/journal.pone.0164577)
Supplement: S2 Appendix — Archive containing the HPLC stability results as browsable html pages. (ZIP) [file pone.0164577.s002.zip › diazoxide_html_results/diazoxide_bottle/index.html?preparation=tablet-oralmixsf&lot=a&condition=bottle-25&time=7.html]

Stability Study Cruncher


### Preparation: tablet-oralmixsf, Lot: a, Condition: bottle-25, Time: 7

Assay (mg/mL): 10.24 ± 0.35 (n = 3);
Assay (%TZ): 100.2 ± 3.4 (n = 3).

| Input String | Area | Cal Id | Cal Slope | Assay | Assay TZ | Assay %TZ |  |
| --- | --- | --- | --- | --- | --- | --- | --- |
| diazoxide\_tablet-oralmixsf\_a\_bottle-25\_7;3883383;;cal7sf200;stability | 3883383 | cal7sf200 | 373260 | 10.40 | 10.22 | 101.8 | calibration, time zero |
| diazoxide\_tablet-oralmixsf\_a\_bottle-25\_7;3909932;;cal7sf200;stability | 3909932 | cal7sf200 | 373260 | 10.48 | 10.22 | 102.5 | calibration, time zero |
| diazoxide\_tablet-oralmixsf\_a\_bottle-25\_7;3674193;;cal7sf200;stability | 3674193 | cal7sf200 | 373260 | 9.84 | 10.22 | 96.3 | calibration, time zero |
