# Supplementary material for: Stability of Diazoxide in Extemporaneously Compounded Oral Suspensions
Source: PLoS One. 2016 Oct 11;11(10):e0164577. doi: 10.1371/journal.pone.0164577 (PMC5058506; doi:10.1371/journal.pone.0164577)
Supplement: S2 Appendix — Archive containing the HPLC stability results as browsable html pages. (ZIP) [file pone.0164577.s002.zip › diazoxide_html_results/diazoxide_bottle/index.html?preparation=tablet-oralmixsf&lot=a&condition=bottle-25&time=60.html]

Stability Study Cruncher


### Preparation: tablet-oralmixsf, Lot: a, Condition: bottle-25, Time: 60

Assay (mg/mL): 10.47 ± 0.91 (n = 3);
Assay (%TZ): 102.5 ± 8.9 (n = 3).

| Input String | Area | Cal Id | Cal Slope | Assay | Assay TZ | Assay %TZ |  |
| --- | --- | --- | --- | --- | --- | --- | --- |
| diazoxide\_tablet-oralmixsf\_a\_bottle-25\_60;3678995;;cal60sf210;stability | 3678995 | cal60sf210 | 358176 | 10.27 | 10.22 | 100.5 | calibration, time zero |
| diazoxide\_tablet-oralmixsf\_a\_bottle-25\_60;4106574;;cal60sf210;stability | 4106574 | cal60sf210 | 358176 | 11.47 | 10.22 | 112.2 | calibration, time zero |
| diazoxide\_tablet-oralmixsf\_a\_bottle-25\_60;3466327;;cal60sf210;stability | 3466327 | cal60sf210 | 358176 | 9.68 | 10.22 | 94.7 | calibration, time zero |
