# Supplementary material for: Stability of Diazoxide in Extemporaneously Compounded Oral Suspensions
Source: PLoS One. 2016 Oct 11;11(10):e0164577. doi: 10.1371/journal.pone.0164577 (PMC5058506; doi:10.1371/journal.pone.0164577)
Supplement: S2 Appendix — Archive containing the HPLC stability results as browsable html pages. (ZIP) [file pone.0164577.s002.zip › diazoxide_html_results/diazoxide_bottle/index.html?preparation=tablet-oralmixsf&lot=a&condition=bottle-25&time=75.html]

Stability Study Cruncher


### Preparation: tablet-oralmixsf, Lot: a, Condition: bottle-25, Time: 75

Assay (mg/mL): 10.89 ± 0.19 (n = 3);
Assay (%TZ): 106.6 ± 1.8 (n = 3).

| Input String | Area | Cal Id | Cal Slope | Assay | Assay TZ | Assay %TZ |  |
| --- | --- | --- | --- | --- | --- | --- | --- |
| diazoxide\_tablet-oralmixsf\_a\_bottle-25\_75;3919509;;cal60sf210;stability | 3919509 | cal60sf210 | 358176 | 10.94 | 10.22 | 107.1 | calibration, time zero |
| diazoxide\_tablet-oralmixsf\_a\_bottle-25\_75;3956081;;cal60sf210;stability | 3956081 | cal60sf210 | 358176 | 11.05 | 10.22 | 108.1 | calibration, time zero |
| diazoxide\_tablet-oralmixsf\_a\_bottle-25\_75;3825929;;cal60sf210;stability | 3825929 | cal60sf210 | 358176 | 10.68 | 10.22 | 104.5 | calibration, time zero |
